# Delivery of mirror image polypeptides into cells†
†Electronic supplementary information (ESI) available. See DOI: 10.1039/c4sc02078b



**DOI:** 10.1039/c4sc02078b

**Published:** 2014-09-25

**Authors:** Amy E. Rabideau, Xiaoli Liao, Bradley L. Pentelute

**Affiliations:** a Department of Chemistry , Massachusetts Institute of Technology , 77 Massachusetts Ave. 18-596 , Cambridge , MA 02139 , USA . Email: blp@mit.edu

## Abstract

Mirror image peptides have unique stability and immunogenic properties in mammals, making them attractive agents to investigate. Their properties inside cells have been mostly unexplored because biopolymers are difficult to transport across cellular membranes. Here, we used protective antigen (PA) from anthrax toxin to deliver mirror image polypeptide cargo into the cytosol of mammalian cells when conjugated to the C-terminus of the PA-binding domain of lethal factor, LF_N_. We found mirror image polypeptides and proteins were translocated as efficiently into cells as their L counterparts. Once in the cytosol, by the use of western blot, we found that d peptides at the C-terminus of LF_N_ were able to achieve higher steady state concentrations when compared to the l-peptide conjugate. With this platform, we delivered a d-peptide MDM2 antagonist to disrupt the p53/MDM2 interaction in cancer cells. For the first time, we show the PA/LF_N_ system is adaptable for the intracellular delivery of mirror image peptides and proteins.

## Abstract 2

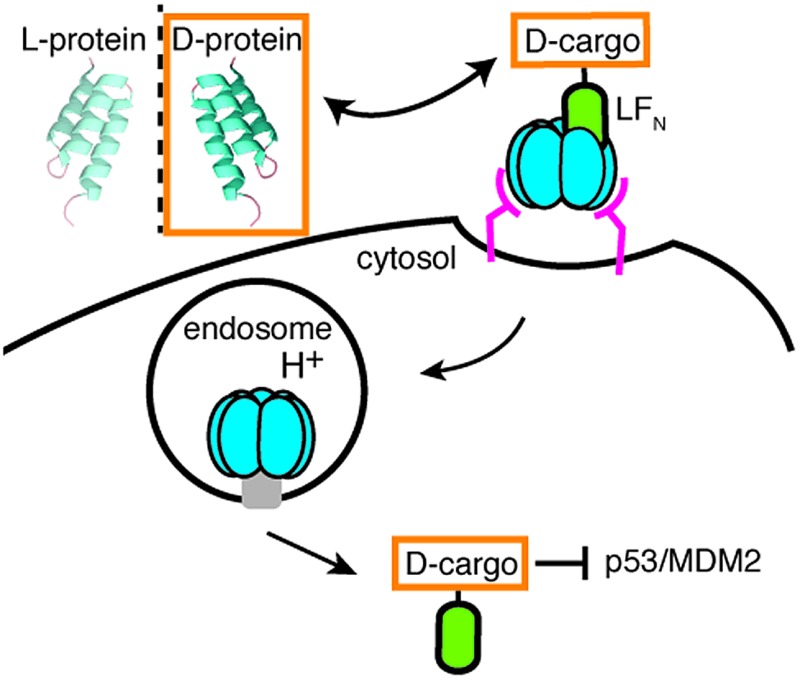
Mirror image peptides have unique stability and immunogenic properties in mammals, making them attractive agents to investigate.

## Introduction

The chirality of biomolecules is critical for their structural integrity and function;^1^ ribosomally produced proteins are composed of l-amino acids and achiral glycine. Polypeptides that contain mirror image or d-amino acids therefore provide a few key advantages over their natural counterparts. Mirror image polypeptides are not recognized by nature's proteases and are thus less susceptible to degradation and have been found to cause low immunogenic response.^2^ To obtain mirror image polypeptides, solid phase peptide synthesis from d-amino acids and sometimes in combination with native chemical ligation are used.^3,4^ Mirror image phage display was developed to generate functional d-polypeptide binders for l-target proteins.^5^

The evolution of bioactive mirror image binders toward intracellular targets has been largely unexplored in part due to the challenge of delivery into the cell cytosol. There are only a few bioactive mirror image peptides that have been delivered inside cells to perturb a biological function, including a d-peptide evolved from mirror image phage display to bind to MDM2 protein^6,7^ or a retro-inverso^8^ peptide that activates the p53 protein. In addition, there are no examples of mirror image proteins delivered into the cell cytosol. Despite extensive efforts in developing intracellular delivery techniques based on cell penetrating peptides (CPPs),^9,10^ cationic lipids,^11^ nanoparticles,^12^ or liposomes,^13^ limited success has been achieved for d-polypeptides.^14^ In the case of d-peptide binders towards MDM2, the fusions to CPPs were nonspecifically cytotoxic in a p53-independent manner, and the most active binders could not be packaged into liposomes for delivery.^6,7^ A system capable of efficient delivery of d-polypeptides into the cell cytosol is of immediate interest since it will allow us not only to investigate the fundamental properties of these modern agents in the cell, but also to use them for perturbation of intracellular processes.

**Fig. 1:**
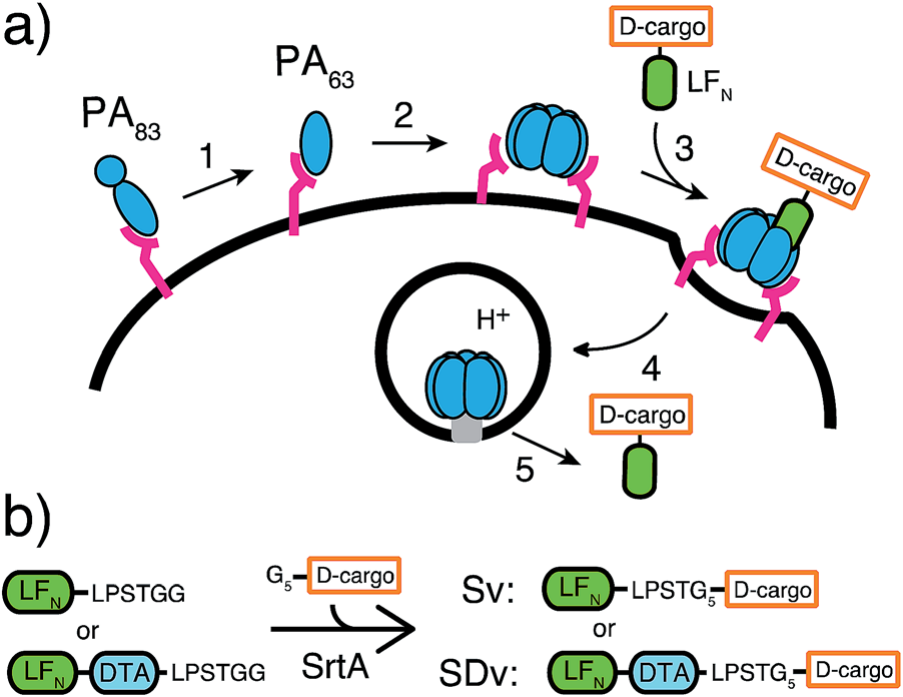
Delivery of d-cargo sortagged onto LF_N_ and LF_N_-DTA. (a) Translocation of d-cargo conjugated to LF_N_ is mediated by protective antigen (PA) from anthrax toxin. (b) G_5_-d-cargo are conjugated to LF_N_ and LF_N_-DTA using SrtA to yield sortagged variants (Sv's) and sortagged DTA variants (SDv's).

Nature has evolved machineries to transport various biomolecules into cells.^15^ One example is *Bacillus anthracis*, which produces anthrax toxin. Anthrax toxin is a three-component system containing protective antigen (PA), a receptor-binding, pore-forming protein, lethal factor (LF)^16^ and edema factor (EF)^17^ as the enzymatic moieties. PA binds to receptors on host cells and is cleaved by furin-family proteases (Fig. 1a, step 1).^18^ The resulting fragment PA_63_ self-assembles into the ring-shaped heptameric^19^ and octameric^20^ pre-pore (Fig. 1a, step 2), forming complexes with LF and EF with high affinity (Fig. 1a, step 3). The complexes are then endocytosed (Fig. 1a, step 4) and endosomal acidification triggers conformational rearrangement of the pre-pore to form an ion-conductive β-barrel transmembrane pore.^21^ The pore then translocates LF and EF into the cytosol to act on their selective target. LF binds stereospecifically to PA pre-pore through the 263-residue N-terminal domain (LF_N_), which initiates the unfolding and translocation of the protein in an N- to C-terminal direction through the narrow β-barrel channel (Fig. 1a, step 5).^22^ Prior work has shown this system can transport cargo into cells but never for mirror image cargo.

Here we combined synthetic chemistry with enzyme-mediated bioconjugation to attach mirror image polypeptides as cargo to the C-terminus of LF_N_. We found that the PA pore can translocate mirror image polypeptide cargo as efficiently as the all l constructs. For the first time, we demonstrated the delivery of intact mirror image protein cargo into the cytosol of eukaryotic cells. We further demonstrated that a mirror image peptide binder delivered by PA was able to disrupt the intracellular p53/MDM2 interaction in cancer cells.

## Results and discussion

We used an evolved sortase A (SrtA*)^23^ for the facile attachment of different d-cargo with an N-terminal oligoglycine motif to the C-terminus of LF_N_ or LF_N_-DTA containing the LPXTG recognition site to give sortagged variants (Sv) or sortagged DTA variants (SDv) (Fig. 1b). LF_N_-DTA contains DTA, which is the A-chain of diphtheria toxin and is extensively used as a reporter of translocation based on its enzymatic activity to ADP ribosylate elongation factor-2 and block protein synthesis once in the cytosol.^24,25^ After confirming the translocation of the LF_N_-DTA variants (SDv's), we investigated delivery into the cytosolic compartment *via* western blot using LF_N_ variants that lack DTA (Sv's).

**Fig. 2:**
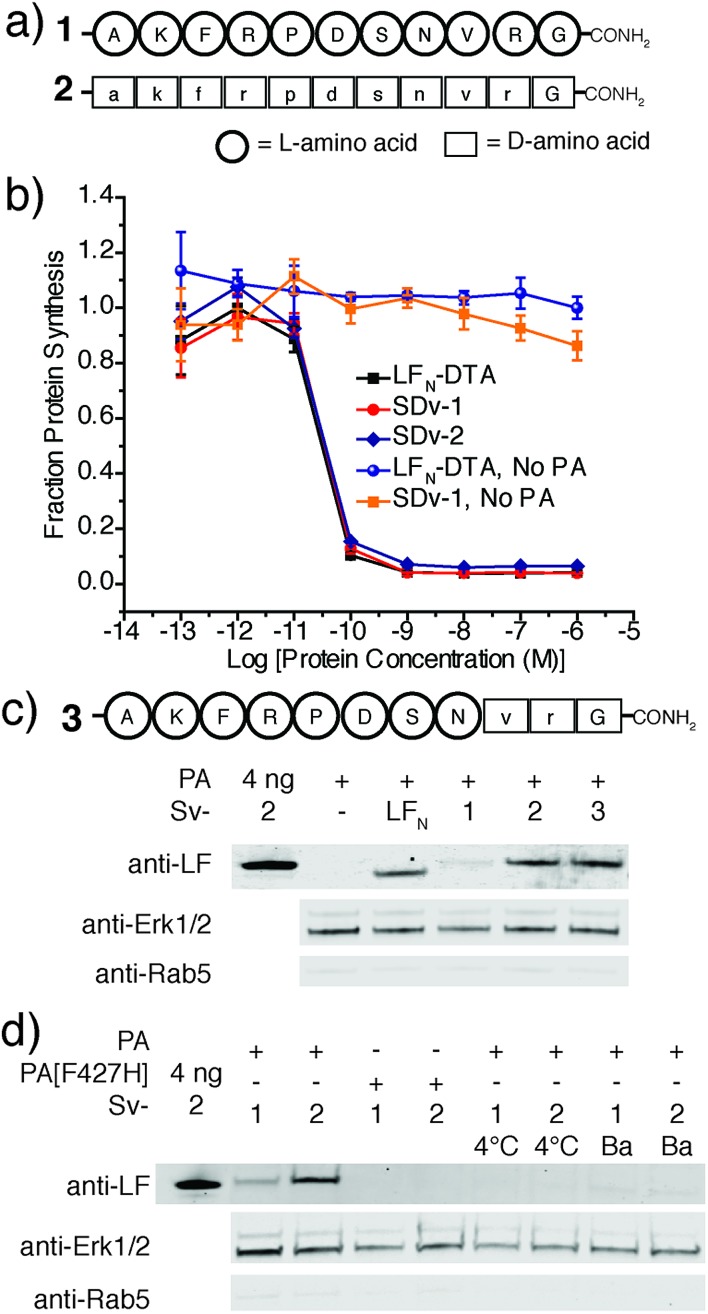
Translocation of mirror peptides using LF_N_/PA (a) Peptide cargo 1 and 2 contain either l or d-amino acids, respectively. (b) Translocation efficiency of SDv1 and SDv2 were analysed using the protein synthesis inhibition assay in CHO-K1 cells treated with varying concentrations of each variant in the presence of 20 nM PA for 30 minutes and protein synthesis was measured using ^3^H-Leu. (c) Peptide cargo 3 and western blot for CHO-K1 treated with 250 nM wild-type LF_N_ or Sv1–3 in the presence of 40 nM PA overnight. (d) CHO-K1 cells were treated with 250 nM Sv1 or Sv2 in the presence of 40 nM PA overnight. The treatment conditions for the control experiments included mutant PA (PA[F427H]), the addition of 200 nM Bafilomycin A1 (Ba), and 4 °C instead of 37 °C incubation (4 °C).

To begin our studies, we synthesized (Table S1†) and sortagged two peptides, (G_5_-AKFRPDSNVRG) one containing all l-amino acids and the other with all d-amino acids, to LF_N_-DTA (Table S2†) to give SDv1 and SDv2 (Tables S3 and S4†), respectively (Fig. 2a). The SDv1 and SDv2 conjugates were added to CHO-K1 cells in the presence of 20 nM PA for 30 minutes, which was sufficient time to allow entry of DTA cargo enzyme into cytosol. After treatment, the cells were washed and incubated with medium supplemented with ^3^H-Leu to determine the efficiency of cargo delivery by measuring protein synthesis (Table S5†). As shown in Fig. 2b, both protein conjugates translocated efficiently, relative to the positive control, LF_N_-DTA, indicating that the PA/LF_N_ system is adaptable for the intracellular delivery of mirror image peptides. Once the unfolding and translocation are initiated by LF_N_ the cargo can ‘piggy-back’ through PA pore regardless of the stereochemistry.

We further analyzed the cytosolic delivery of the LF_N_ variants Sv1 and Sv2 by western blot. CHO-K1 cells were incubated with Sv1 and Sv2 in the presence of PA overnight to allow for multiple rounds of receptor mediated endocytosis and translocation. Cells were washed, trypsin digested to remove cell surface receptors and bound proteins, and then extracted with buffer containing 50 μg mL^–1^ digitonin. Digitonin is a mild detergent used to permeabilize the plasma membrane and to extract only the cytosolic fraction.^26^ Immunoblotting with antibodies against Rab5 (an early endosome marker) and Erk1/2 (a cytosolic protein) is used to validate the extraction of cytosolic proteins. Immunoblot analysis using anti-LF antibody revealed that both conjugates translocated into cells and Sv2 reached a similar steady state concentration to LF_N_; however for Sv1, we observed a significantly less intense band than Sv2 (Fig. 2c). Since the delivery efficiencies were the same for all three constructs from the protein synthesis inhibition assay, the steady state levels in the cell could reflect the difference in extracellular or intracellular stability. We observed similar stability in serum-containing medium for Sv1, Sv2, and LF_N_ (Fig. S1†), we therefore hypothesize that the peptides used here acted as unstructured cargo on the C-terminus of LF_N_, and facilitated degradation. In this case, the l-peptide promoted degradation while the d-peptide did not. We investigated this observation with a variant containing the l-peptide capped with two d-amino acids at the C-terminus (Sv3). We observed similar concentration of Sv3 in the cytosol as wild-type LF_N_, indicating the C-terminal d-amino acids played an essential role in the variant's intracellular stability. These observations suggest that a d-peptide cap can possibly promote stabilization inside the cell.

In order to confirm the translocation mechanism of Sv1 and Sv2, we performed three control experiments and monitored their translocation by western blot (Fig. 2d). We first incubated the cells at 4 °C instead of 37 °C, which inhibited translocation of both Sv1 and Sv2, indicating that endocytosis is necessary for translocation. Next, we found that treatment with 200 nM Bafilomycin A1, a vacuolar H^+^-ATPase inhibitor, prevented translocation, indicating the importance of an acidic endosome. Finally, we used a PA mutant, PA[F427H], that binds LF_N_ and is endocytosed but arrests translocation. We observed no cargo in the cytosol when cells were incubated with PA[F427H] instead of PA, indicating that Sv1 and Sv2 must pass through the translocase for entry into the cytosol. These results confirmed that the translocation of Sv1 and Sv2 followed the same mechanism as LF_N_.

**Fig. 3:**
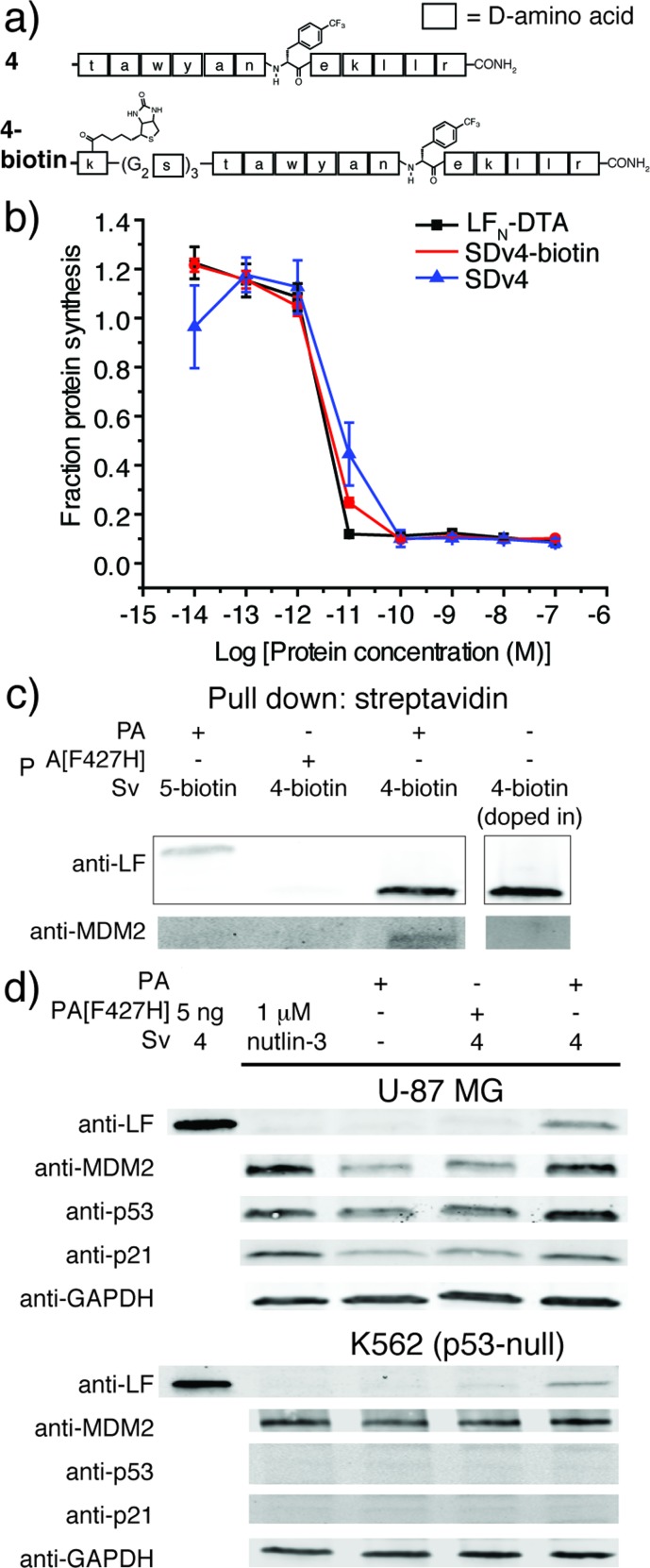
Translocation of a d-binder to MDM2. (a) Structure of a d-peptide binder to MDM2 (4) and its biotinylated form (4-biotin). (b) Translocation efficiency of SDv4 and SDv4-biotin were analysed using the protein synthesis inhibition assay in CHO-K1 cells treated with varying concentrations of each variant in the presence of 20 nM PA for 2 hours. (c) U-87 MG cells were treated with 150 nM Sv4-biotin in the presence of 20 nM PA for 24 h. The cell lysates were then treated with streptavidin beads to co-precipitate Sv4-biotin and MDM2. Sv5-biotin with PA and Sv4-biotin with PA[F427H] served as negative controls. An additional negative control was run where 5 nM Sv4-biotin was doped into cell lysate then treated to the same pull down procedure. (d) Western blot analysis of U-87 MG cells treated with 150 nM Sv4 in the presence of 20 nM PA or PA[F427H] or 1 μM nutlin-3 for 24 h. As a negative control, K562 (p53-null) cells were analysed under the same conditions.

We next explored PA mediated delivery of a bioactive d-peptide to disrupt an intracellular protein–protein interaction. We chose a d-peptide (TAWYANF*EKLLR, where F* is *p*-CF_3_-d-Phe) that has a *K*_d_ of 0.45 nM towards MDM2.^7^ Prior work with variants of this peptide proved challenging as fusions to CPPs were toxic and the most active binders could not be packaged into liposomes for delivery. To study PA mediated delivery of the d-peptide binder into the cytosol of human glioblastoma (U87-MG) cells, we sortagged the peptide and its biotinylated form onto LF_N_-DTA to give SDv4 and SDv4-biotin, respectively (Fig. 3a). Both variants translocated equally well based on the protein synthesis inhibition assay (Fig. 3b). We then studied the cellular function of the d-peptide binder by sortagging the peptide and its biotinylated form onto LF_N_ to give Sv4 and Sv4-biotin, avoiding the interference of DTA toxicity to the cell. Both constructs were delivered and detected by western blot in U-87 MG cells. Based on the linear relationship between the amount of protein loaded and the band intensity detected by anti-LF antibody, we estimated a total of 0.9 ng Sv4 delivered into 76 000 cells, giving 250 000 molecules per cell or 110 nM of cytosolic concentration (Fig. 3d and S2†).

We investigated whether the attachment of LF_N_ would affect the interaction between the peptide and MDM2. We expressed ^25-109^MDM2 as a SUMO fusion (SUMO-^25-109^MDM2) and used this construct to measure the binding affinity for cargo 4 and its LF_N_ conjugate Sv4 using a bilayer interferometry system (Fig. S3 and S4†). Both cargo 4 and Sv4 effectively competed with immobilized biotin-^15-29^p53 for SUMO-^25-109^MDM2 binding, yielding *K*_d_ values of 1.0 ± 0.7 nM and 12.3 ± 4.3 nM, respectively. Despite the reduction in binding affinity as compared to the cargo 4 by itself, Sv4 still had low nanomolar affinity for MDM2.

We analyzed whether the delivered peptide could interact with MDM2 in the cell cytosol. Streptavidin agarose beads were used to capture Sv4-biotin from the lysate of cells treated with Sv4-biotin and PA. We then eluted the bound proteins from the beads and analyzed by immunoblotting with anti-LF and anti-MDM2 antibody. As shown in Fig. 3c, we observed bands corresponding to Sv4-biotin and the bound MDM2 from the lysate of cells treated with Sv4-biotin and PA, whereas no MDM2 was detected in the control experiments when PA[F427H] was used instead of PA, or a MDM2 non-binding control (Sv5-biotin, Fig. S5†) was used instead of Sv4-biotin. These results show the specific binding between delivered Sv4-biotin and intracellular MDM2. To confirm that the binding event occurred inside the cell but not post-lysis, we added a similar amount of Sv4-biotin directly into the U-87 MG cell lysate, and no MDM2 was detected in the pull-down fraction. The absence of MDM2 pull down in this control experiment confirms that the binding between delivered peptide and MDM2 occurred in the cytosol.

Next, we studied the biological effect of the binding between Sv4 and MDM2 inside U-87 MG cells. Binding an antagonist in the p53-binding domain of MDM2 disrupts this interaction and results in activation and expression of MDM2, p53, and p21.^27^ Therefore, we quantified the relative change in protein levels of p53, MDM2, and p21 in U-87 MG cells under different treatment conditions, and used the small molecule MDM2 antagonist nutlin-3 as a positive control. By immunoblotting, as compared to the PA only or PA[F427H] conditions, we observed increased levels of MDM2, p53, and p21 for U-87 MG cells treated with Sv4 and PA (Fig. 3d), similar to the cells treated with 1 μM nutlin-3. In order to confirm that this upregulation is dependent on the disruption of p53/MDM2 interaction, we performed the same analysis on the K562 leukemia cell line, which lacks p53.^28^ Despite the delivery of Sv4 into K562 cells, we observed no perturbation of MDM2 and p21 protein levels. These findings strongly support Sv4 regulated protein levels by specifically inhibiting the p53/MDM2 interaction. Taken together, our results indicate a d-peptide can be delivered to the cytosol of U-87 MG cancer cells and reach a concentration sufficient to bind the target protein and disrupt a critical protein–protein interaction.

**Fig. 4:**
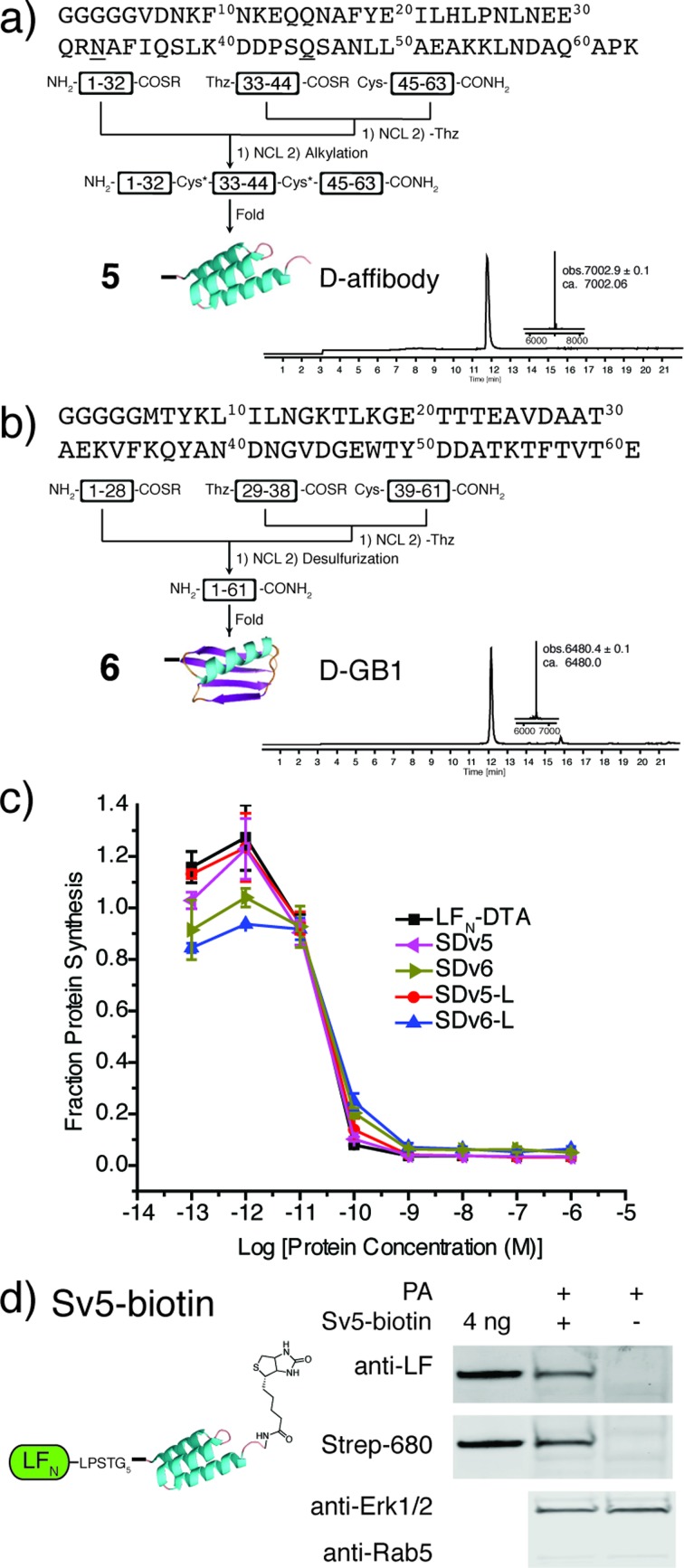
Synthesis and translocation of mirror image proteins. (a) Synthetic scheme for d-affibody (5) and LC-MS analysis of d-affibody (TIC with deconvoluted mass inset). (b) Synthetic scheme for d-GB1 (6) and LC-MS analysis of d-GB1 (TIC with deconvoluted mass inset). (c) Translocation efficiency of SDv5 and SDv6 were analysed using the protein synthesis assay in CHO-K1 cells treated with varying concentrations of each variant in the presence of 20 nM PA for 30 min and compared to the efficiencies of l-affibody (SDv5-l) and l-GB1 (SDv6-l) were also analysed. (d) CHO-K1 cells were treated with 100 nM Sv5-biotin in the presence of 40 nM PA for 24 hours and analysed after digitonin extraction with anti-LF antibody and streptavidin-IRDye 680.

After demonstrating that PA-mediated translocation of mirror image peptides can be efficiently delivered, we next investigated the possibility of using PA to translocate mirror image proteins a feat never accomplished before. We chose to study two mirror image proteins, d-affibody containing three α-helices^29^ and d-protein GB1 containing α-helices and a β-sheet.^30^ We chemically synthesized these proteins from three fragments (Fig. S6 and S7†) using *in situ* neutralization Boc SPPS.^31^ After cleavage and purification of each fragment, the peptide segments were ligated together using native chemical ligation, according to the synthetic strategies in Fig. 4a and b.^4^ The cysteine residues of d-affibody were alkylated to give pseudo-glutamine within the full-length protein, while the cysteine residues of d-GB1 were desulfurized^32^ to give the native sequence. The mirror image conformation of the folded proteins was evidenced by circular dichroism (CD) spectra (Fig. S8†). An *in vitro* tryptic digest of l- and d-affibody was performed to measure the stability of the proteins over time (Fig. S9†). Based on LC-MS analysis, we observed complete degradation of the l-affibody and no degradation of the d-affibody. This demonstrates that mirror image polypeptides are less susceptible to proteolytic degradation. When conjugated to LF_N_, we found that LF_N_-d-affibody (Sv5) was more stable than the all l-protein LF_N_-l-affibody towards trypsin digestion, indicating a unique property of the hybrid protein.

To study translocation efficiency, the d-proteins were sortagged onto LF_N_-DTA to give SDv5 and SDv6, respectively. As evidenced by the DTA-mediated inhibition of protein synthesis, both mirror image variants translocated as efficiently as LF_N_-DTA (Fig. 4c). In order to confirm that d-affibody was delivered to the cytosol as an intact protein, we studied the translocation of LF_N_ conjugates of d-affibody (Sv5) and incorporated an alkyne functional group at the C-terminus of d-affibody for functionalization with azide-biotin to yield Sv5-biotin (Fig. S10†). After translocation into CHO-K1 cells, the digitonin-extracted cytosolic fraction was immunoblotted with anti-LF antibody and showed Sv5-biotin delivery into the cytosol (Fig. 4d). Delivery of Sv5-biotin was further confirmed by staining with streptavidin conjugated to an IR680 dye, which revealed a similar amount to that detected by anti-LF antibody (Fig. 4d). For the first time we show the delivery of intact mirror image proteins into the cytosol by PA, thereby facilitating the investigation of their biological properties inside the cell.

## Conclusions

In this work, we showed that once translocation is initiated by LF_N_, the interaction between the PA pump and cargo attached at the C-terminus of LF_N_ is independent of the stereochemistry. It was not clear that the PA/LF_N_ system would allow for the delivery since most protein–protein interactions are stereospecific and disruption of chirality results in loss of function. The proper function of PA/LF_N_ is dependent on stereospecific interactions that are required for binding and initiation of translocation; however, we demonstrated that the interaction between the PA translocase and cargo is not necessarily stereospecific. In this study, we used two assays, the protein synthesis inhibition assay based on DTA activity and western blot of cytosolic proteins, and found that PA efficiently translocated mirror image cargo into the cell cytosol.

We found that translocation of d-cargo is not limited to the length or fold of the polypeptide. We demonstrated the translocation of two mirror image proteins: affibody and GB1. We further confirmed delivery of an intact mirror image protein by staining the translocated biotinylated d-affibody with anti-LF antibody and fluorescently labeled streptavidin. However, we currently have no tools to assess their folding or activity inside the cytosol. Based on the protein synthesis inhibition data, the foreign protein DTA correctly refolds in the cytosolic environment. Our future efforts to generate bioactive mirror image proteins will give us the capability to assay their intracellular function and provide insight into refolding after translocation into the cell.

We further demonstrated that the PA/LF_N_ system efficiently delivers functional d-peptides for the disruption of intracellular protein–protein interaction in cancer cells. We focused on a mirror image peptide that was previously reported a challenge to deliver into cells.^6,7^ Our own efforts to load this peptide into liposomes for delivery failed. In contrast, for the first time we found that the PA/LF_N_ efficiently delivered this d-peptide. We found it bound to its target after reaching the cytosol, as confirmed by capture assays and western blot. Furthermore, the d-binder perturbed the p53/MDM2 interaction and activated related pathway. The delivery problem has been the major obstacle to place mirror image polypeptides in the cytosol of cells. Using the SrtA conjugation and the PA/LF_N_ delivery platform, we overcame this challenge.

## References

[cit1] Milton R. C. D., Milton S. C. F., Kent S. B. H. (1992). Science.

[cit2] Dintzis H. M., Symer D. E., Dintzis R. Z., Zawadzke L. E., Berg J. M. (1993). Proteins: Struct., Funct., Genet..

[cit3] Pentelute B. L., Gates Z. P., Dashnau J. L., Vanderkooi J. M., Kent S. B. H. (2008). J. Am. Chem. Soc..

[cit4] Dawson P. E., Muir T. W., Clark-Lewis I., Kent S. B. H. (1994). Science.

[cit5] Schumacher T. N. M., Mayr L. M., Minor D. L., Milhollen M. A., Burgess M. W., Kim P. S. (1996). Science.

[cit6] Liu M., Li C., Pazgier M., Li C. Q., Mao Y. B., Lv Y. F., Gu B., Wei G., Yuan W. R., Zhan C. Y., Lu W. Y., Lu W. Y. (2010). Proc. Natl. Acad. Sci. U. S. A..

[cit7] Zhan C., Zhao L., Wei X., Wu X., Chen X., Yuan W., Lu W.-Y., Pazgier M., Lu W. (2012). J. Med. Chem..

[cit8] Li C., Zhan C., Zhao L., Chen X., Lu W.-Y., Lu W. (2013). Bioorg. Med. Chem..

[cit9] Caron N. J., Torrente Y., Camirand G., Bujold M., Chapdelaine P., Leriche K., Bresolin N., Tremblay J. P. (2001). Mol. Ther..

[cit10] Heitz F., Morris M. C., Divita G. (2009). Br. J. Pharmacol..

[cit11] Zelphati O., Wang Y., Kitada S., Reed J. C., Felgner P. L., Corbeil J. (2001). J. Biol. Chem..

[cit12] Gu Z., Biswas A., Zhao M. X., Tang Y. (2011). Chem. Soc. Rev..

[cit13] Torchilin V. P., Rammohan R., Weissig V., Levchenko T. S. (2001). Proc. Natl. Acad. Sci. U. S. A..

[cit14] Foerg C., Merkle H. P. (2008). J. Pharm. Sci..

[cit15] The Comprehensive Sourcebook of Bacterial Protein Toxins, ed. J. E. Alouf and M. R. Popoff, Academic Press, 2006.

[cit16] Pannifer A. D., Wong T. Y., Schwarzenbacher R., Renatus M., Petosa C., Bienkowska J., Lacy D. B., Collier R. J., Park S., Leppla S. H., Hanna P., Liddington R. C. (2001). Nature.

[cit17] Leppla S. H. (1982). Proc. Natl. Acad. Sci. U. S. A..

[cit18] Klimpel K. R., Molloy S. S., Thomas G., Leppla S. H. (1992). Proc. Natl. Acad. Sci. U. S. A..

[cit19] Milne J. C., Furlong D., Hanna P. C., Wall J. S., Collier R. J. (1994). J. Biol. Chem..

[cit20] Kintzer A. F., Thoren K. L., Sterling H. J., Dong K. C., Feld G. K., Tang I. I., Zhang T. T., Williams E. R., Berger J. M., Krantz B. A. (2009). J. Mol. Biol..

[cit21] Krantz B. A., Trivedi A. D., Cunningham K., Christensen K. A., Collier R. J. (2004). J. Mol. Biol..

[cit22] Arora N., Leppla S. H. (1993). J. Biol. Chem..

[cit23] Chen I., Dorr B. M., Liu D. R. (2011). Proc. Natl. Acad. Sci. U. S. A..

[cit24] Collier R. J., Kandel J. (1971). J. Biol. Chem..

[cit25] Wilson B. A., Collier R. J. (1992). Curr. Top. Microbiol. Immunol..

[cit26] Adam S. A., Marr R. S., Gerace L. (1990). J. Cell Biol..

[cit27] Eldeiry W. S., Tokino T., Velculescu V. E., Levy D. B., Parsons R., Trent J. M., Lin D., Mercer W. E., Kinzler K. W., Vogelstein B. (1993). Cell.

[cit28] Law J. C., Ritke M. K., Yalowich J. C., Leder G. H., Ferrell R. E. (1993). Leuk. Res..

[cit29] Lofblom J., Feldwisch J., Tolmachev V., Carlsson J., Stahl S., Frejd F. Y. (2010). FEBS Lett..

[cit30] Mandal K., Uppalapati M., Ault-Riche D., Kenney J., Lowitz J., Sidhu S. S., Kent S. B. H. (2012). Proc. Natl. Acad. Sci. U. S. A..

[cit31] Schnolzer M., Alewood P., Jones A., Alewood D., Kent S. B. H. (1992). Int. J. Pept. Protein Res..

[cit32] Wan Q., Danishefsky S. J. (2007). Angew. Chem.,Int. Ed..

