# Supplementary material for: Delivery of mirror image polypeptides into cells
Source: Chem Sci. 2014 Sep 25;6(1):648–53. doi: 10.1039/c4sc02078b (PMC5492103; doi:10.1039/c4sc02078b)
Supplement: Supplementary file 1 [file SC-006-C4SC02078B-s001.pdf]

Electronic Supplementary Material (ESI) for Chemical Science.
This journal is © The Royal Society of Chemistry 2014

Delivery of Mirror Image Polypeptides into Cells

Amy E. Rabideau,[+] Xiaoli Liao, [+] Bradley L. Pentelute*

Supplementary Information

Table of Contents

MATERIALS		3
METHODS	Solid phase peptide synthesis (Boc)	3
	Solid phase peptide synthesis (Fmoc)	3
	Analytical LC-MS	4
	Preparative, semi-preparative, and analytical RP-HPLC	4
	Synthesis of D-affibody	4
	Synthesis of D-affibody-alkyne	5
	Synthesis of D-GB1	5
	Circular dichroism (CD) spectroscopy of folded proteins	6
	Synthesis of biotinylated p53/MDM2 Inhibitor D-peptide	6
	Construction of plasmids for recombinant proteins	6
	Protein expression and purification	7
	One-pot sortagging reaction using *Staphylococcus aureus* SrtA*	7
	Translocation of SDv's and protein inhibition assay in CHO-K1 cells	8
	Cytosolic protein extraction and whole cell lysate preparation for western blot	8
	Western Blot of Sv1, 2, 4, and 5 translocated into CHO-K1 cells	8
	Trypsin digestion of L- and D-Affibody	8
	Pull down of Sv4-biotin in U-87 MG cells	9
	Translocation of Sv4 and Sv4-biotin in U-87 MG or K562 cells	9
	Binding interaction between SUMO-$^{25\text{-}109}$MDM2 and 4 or Sv4	9
TABLES	Table S1. Peptides used in this investigation	11
	Table S2. Observed molecular masses of expressed protein constructs when analyzed by LC-MS	12
	Table S3. List of variants	13
	Table S4. Isolated yields of sortagging ligations from SrtA* reaction	14
	Table S5. EC50 values of 30-minute protein synthesis inhibition assay	15
FIGURES	Figure S1. Immunoblot of media from CHO-K1 cells treated with Sv1-3	16
	Figure S2. Linear relationship between Sv4 band intensity and the amount of protein loaded	17
	Figure S3. Calibration curve for the interaction between immobilized biotin-$^{15\text{-}29}$p53 and SUMO-$^{25\text{-}109}$MDM2	18
	Figure S4. Binding interaction between SUMO-$^{25\text{-}109}$MDM2 and 4 or Sv4 based on a competition binding assay at 30 °C	19
	Figure S5. Quantification of MDM2, p53, and p21 protein levels for U-87 MG cells	20
	Figure S6. LC-MS characterization of D-affibody synthesis	21
	Figure S7. LC-MS characterization of D-GB1 synthesis	22
	Figure S8. CD of L- and D-affibody and L- and D-GB1	23
	Figure S9. Trypsin digestion of L-affibody (A) and D-affibody (B) at t=0, 3, and 18 h and Sv5-L (C) and Sv5-alkyne at t=0, 9, and 23 h.	24-5
	Figure S10. LC-MS characterization of D-affibody-alkyne and -biotin	26
LC-MS appendix		27
References		30

MATERIALS

2-(1H-Benzotriazol-1-yl)-1,1,3,3-tetramethyluronium hexafluorophosphate (HBTU), N-α-Boc, and N-α-Fmoc protected L- and D-amino acids were purchased from Chem-Impex International, IL, Peptide Institute, Japan, and Midwest Bio-Tech, Inc., IN. 4-Methylbenzhydrylamine (MBHA) resin was obtained from Anaspec, CA. N,N-dimethylformamide (DMF), dichloromethane (DCM), methanol (MeOH), diethyl ether, HPLC-grade acetonitrile (MeCN), and guanidine hydrochloride (guanidine·HCl) were from VWR, PA. Trifluoroacetic acid (TFA) was purchased from NuGenTec, CA and Halocarbon, NJ. 2,2'-Azobis[2-(2-imidazolin-2-yl)propane]dihydrochloride (VA-044) was purchased from Wako Pure Chemical Industries, Ltd., Japan. All other reagents were purchased from Sigma-Aldrich, MO and Life Technologies, CA.

The following primary and secondary antibodies were used goat anti-LF (bD-17, Santa Cruz Biotechnology, TX), rabbit anti-MDM2 (N-20, Santa Cruz Biotechnology, TX), rabbit anti-p21 (C-21, Santa Cruz Biotechnology, TX), mouse anti-p53 (DO-1, Santa Cruz Biotechnology, TX), rabbit anti-GAPDH (Sigma Aldrich, MO), goat anti-mouse IRdye 680RD (LI-COR Biosciences, NE), goat anti-rabbit IRdye 800CW (LI-COR Biosciences, NE), and donkey anti-goat IRdye 680LT (LI-COR Biosciences, NE).

METHODS

Solid phase peptide synthesis (Boc)

Select peptides were synthesized on a 0.2 mmol scale on MBHA resin using *in situ* neutralization Boc chemistry protocols.[1] Peptide thioesters were prepared using a S-trityl mercaptopropionic acid (MPA) strategy.[2] Side-chain protection for L- and D-amino acids included: Arg(Tos), Asn(Xan), Asp(OcHex), Cys(4-MeBzl), Glu(OcHex), His(Bom), Lys(2-ClZ), Lys(Alloc), Ser(Bzl), Thr(Bzl), Trp(CHO), and Tyr(2-BrZ). After completion of stepwise SPPS, peptides containing Trp were subjected to 10% piperidine and 5% H_2O (v/v) in DMF for 2 h at RT to remove the formyl protecting group prior to final deprotection of Boc group.

The allyloxycarbonyl (Alloc) protecting group was removed using 4.85 mmol phenylsilane and 39.5 μmol tetrakis(triphenylphosphine)palladium(0) in DCM for 20 min at RT.[3] The resin was washed with DCM then DMF.

The peptides were simultaneously cleaved from the resin and side-chains were deprotected by treatment with 10% (v/v) *p*-thiocresol and 10% (v/v) *p*-cresol in anhydrous HF for 1 h at 0 °C. Peptides were triturated with cold diethyl ether, dissolved in 50:50 (v/v) H_2O: MeCN containing 0.1% TFA (v/v) and lyophilized. The solvent compositions used in the experiments will be referred to as A: 0.1 % TFA in H_2O (v/v), B: 0.1 % TFA in MeCN (v/v).

Solid phase peptide synthesis (Fmoc)

Select peptides were synthesized on a 0.1 mmol scale on aminomethyl resin with a Rink amide linker using fast flow Fmoc synthesis.[4] Side-chain protection for the D-amino acids (Chem-Impex International) included: Arg(Pbf), Asn(Trt), Glu(OtBu), Lys(Boc), Lys(Alloc), Ser(tBu), Thr(tBu), Trp(Boc), and Tyr(tBu). After synthesis, peptides were cleaved from resin with 94% TFA containing 2.5% EDT, 2.5% H_2O, and 1% TIPS (v/v) for 2 h at RT. After cleavage, TFA was dried under N_2(g) and triturated three times with cold ether then dissolved in 50:50 A:B and lyophilized. Purification of the crude peptides was achieved using RP-HPLC.

Analytical LC-MS

All peptides, proteins and semi-synthetic products were analyzed on an Agilent 6520 Accurate-Mass quadrupole time-of-flight (Q-TOF) liquid chromatography-mass spectrometry (LC-MS) system. Solvent A' 0.1% formic acid in H_2O and solvent B': 0.1% formic acid in MeCN was used. LC-MS used Agilent Zorbax 300SB C_3 column (2.1 x 150 mm, 5 μm) using a linear gradient of 5-65% B' over 15 min at a flow rate of 0.4 mL/min. The observed mass was generated by averaging the major peak in the total ion current (TIC). The charge-state series of the species were deconvoluted using Agilent MassHunter Bioconfirm using maximum entropy setting.

Preparative, semi-preparative, and analytical RP-HPLC

The crude peptides were dissolved in 99:1 or 95:5 A:B. If the peptide was insoluble under these solvent conditions, 6M guanidine·HCl was added to the solution. Peptides were purified by preparative RP-HPLC on Agilent Zorbax SB C_{18} column (21.2 x 250 mm, 7 μm) at a flow rate of 10 mL/min at 1-41% B, 5-45% B, or 10-50% B over 80 min. For semi-preparative RP-HPLC, a Agilent Zorbax 300SB C_{18} column (9.4 x 250 mm, 5 μm) was used at a flow rate of 5 mL/min over the same gradients. HPLC fractions were spotted with MALDI matrix alpha-cyano-4-hydroxycinnamic acid (α-CHCA) in 50:50 A:B and checked for the correct molecular masses. The analytical RP-HPLC Agilent C_{18} Zorbax SB column (2.1 x 150 mm, 5 μm) was used to confirm the purity of fractions at a flow rate of 0.5 mL/min over a linear gradient of 1-51% B over 12 min. Analytical HPLC UV absorbance traces were measured at 214 nm.

Synthesis of D-affibody

The synthesis of D-affibody containing G_5 at the N-terminus (63-mer) was performed by native chemical ligation (NCL) of three peptide segments (Scheme S1).

The peptide segments (and the corresponding masses) used in this synthesis were as follows (X: -S-CH_2-CH_2-CO-, Z: thiazolidine):

[Gly^1–Arg^{32}]-$^{\alpha}$thioester: GGGGGVDNKFNKEQQNAFYEILHLPNLNEEQRXA
(calc. average: 3773.8 Da, obs. 3773.8 ± 0.1)
[Thz^{33}–Ser^{44}]-$^{\alpha}$thioester: ZAFIQSLKDDPSXA
(calc. monoisotopic: 1493.7, obs: 1493.7 ± 0.1)
[Cys^{45}–Lys^{63}]-$CONH_2$: CSANLLAEAKKLNDAQAPK
(calc. monoisotopic: 1983.1 Da, obs: 1983.1 ± 0.1)

Purification of peptide segments All three segments were dissolved in 99:1 A:B and purified by preparative RP-HPLC. Fractions containing the purified fragments were combined and lyophilized: [Gly^1–Arg^{32}]-$^{\alpha}$thioester 43.1 mg (10.2 μmol), [Thz^{33}–Ser^{44}]-$^{\alpha}$thioester 118.7 mg (68.9 μmol) and [Cys^{45}–Lys^{63}]-$CONH_2$ 152.1 mg (62.3 μmol).

NCL of three peptide segments Peptide fragments [Thz^{33}–Ser^{44}]-$^{\alpha}$thioester (11.0 mg, 6.4 μmol) and [Cys^{45}–Lys^{63}]-$CONH_2$ (14.0 mg, 5.8 μmol) were dissolved to a concentration of 4 mM in NCL buffer (6 M guanidine·HCl, 20 mM TCEP·HCl, and 40 mM MPAA in 0.2 M sodium phosphate buffer) (Figure S1A) at pH 6.95 at RT for 7 h. $MeONH_2$·HCl was then added to the crude reaction mixture at a final concentration of 0.2 M and pH 4.0 at RT overnight to give [Cys^{33}–Ser^{44}]-[Cys^{45}–Lys^{63}]-$CONH_2$ (Figure S1B). In the same pot, [Gly^1–Arg^{32}]-$^{\alpha}$thioester (26.6 mg, 6.3 μmol) was added (Figure S1C) and incubated at pH 7.0 at RT for 7 h to give [Gly^1–Arg^{32}]-[Cys^{33}–Ser^{44}]-[Cys^{45}–Lys^{63}]-$CONH_2$ (Figure S1D). The product was purified by

semi-preparative RP-HPLC and fractions containing the pure product were combined and lyophilized to give 11.7 mg, 1.5 μmol (26% yield).

Alkylation [Gly^1–Arg^{32}]-[Cys^{33}–Ser^{44}]-[Cys^{45}–Lys^{63}]-$CONH_2$ The two cysteine positions of [Gly^1–Arg^{32}]-[Cys^{33}–Ser^{44}]-[Cys^{45}–Lys^{63}]-$CONH_2$ were alkylated in 6 M guanidine·HCl, 20 mM TCEP·HCl, and 50 mM 2-bromoacetamide in 0.2 M sodium phosphate buffer. The reaction was incubated at pH 7.1 for 30 minutes at RT and quenched with MESNa (100 mM final concentration) to give [Gly^1–Lys^{63}]-$CONH_2$. The product was purified by semi-preparative RP-HPLC and fractions containing pure product were combined and lyophilized to give 9 mg, 1.1 μmol (76% yield) (Figure S1E).

Folding of [Gly^1–Lys^{63}]-$CONH_2$ Full-length [Gly^1–Lys^{63}]-$CONH_2$ was dissolved in 6 M guanidine·HCl, 20 mM Tris-HCl, and 150 mM NaCl, pH 8.5, and was diluted from 6 M to 2 M guanidine·HCl using the same buffer without guanidine·HCl. The peptide solution was desalted into 20 mM Tris-HCl, 150 mM NaCl, pH 8.5 using a HiTrap Desalting column. The folded protein was concentrated using a 3 kDa concentrator to give 5.5 mg, 0.79 μmol (69% yield) (Figure S1F).

Synthesis of D-affibody-alkyne

D-affibody-alkyne was synthesized using the same strategy with an additional propargyl-glycine incorporated at the C-terminus of the protein. D-affibody-biotin was obtained by Cu(I)-catalyzed azide-alkynyl click reaction to label the D-affibody-alkyne with biotin-azide. For the labeling reaction, 50 μL D-affibody-alkyne (332 μg, 46.8 nmol) was mixed with final concentration of 100 mM Tris pH 8.5, 1 mM biotin-azide (70 nmol), 2 mM $CuSO_4$ and 200 mM ascorbic acid in a total 70 μL reaction. LC-MS analysis showed 80% yield after 4 hours reaction at room temperature. The reaction mixture was dissolved in 6 M guanidine·HCl, 20 mM Tris-HCl, and 150 mM NaCl, pH 8.5, and was serially diluted from 6 M to 3 M then to 1 M guanidine·HCl using the same buffer without guanidine·HCl. The solution was desalted into 20 mM Tris-HCl, 150 mM NaCl, pH 7.5 using a HiTrap Desalting column. The folded protein was concentrated using a 3 kDa concentrator to give 202 μg, 28.9 nmol (61% yield).

Synthesis of D-GB1

The synthesis of D-GB1 containing G_5 at the N-terminus (61-mer) was performed by NCL of three peptide segments (Scheme S2).

The peptide segments (and the corresponding masses) used in this synthesis were as follows (X: -S-CH_2-CH_2-CO-, Z: thiazolidine):

[Gly^1–Ala^{28}]-$^{\alpha}$thioester: GGGGGMTYKLILNGKTLKGETTTEAVDAXA
(calc. monoisotopic: 2940.4 Da, obs: 2940.5 ± 0.1 Da)
[Thz^{29}–Tyr^{38}]-$^{\alpha}$thioester: ZTAEKVFKQYXA
(calc. monoisotopic: 1387.6 Da, obs: 1387.6 ± 0.1 Da)
[Cys^{39}–Glu^{61}]-$CONH_2$: CNDNGVDGEWTYDDATKTFTVTE
(calc. monoisotopic: 2579.0 Da, obs: 2579.1 ± 0.1 Da)

Purification of peptide segments All segments were dissolved in 6 M guanidine·HCl in 95:5 A:B and purified by preparative RP-HPLC. Peptide segments [Gly^1–Ala^{28}]-$^{\alpha}$thioester and [Cys^{39}–Glu^{61}] were further purified by semi-preparative RP-HPLC. Fractions containing the purified segments were combined and lyophilized: [Gly^1–Ala^{28}]-$^{\alpha}$thioester 34.3 mg (11.65

μmol), [Thz^{29}–Tyr^{38}]-$^{\alpha}$thioester 57.3 mg (41.3 μmol), and [Cys^{39}–Glu^{61}] 40.9 mg (15.8 μmol).

NCL of three peptide segments Peptide segments [Thz^{29}–Tyr^{38}]-$^{\alpha}$thioester (13.2 mg, 9.5 μmol) and [Cys^{39}–Glu^{61}] (20.5 mg, 7.9 μmol) were dissolved to a concentration of 4 mM in NCL buffer (Figure S3A). The reaction was incubated at pH 7.0 at RT for 3 h. $MeONH_2$·HCl was then added to the crude reaction mixture at a final concentration of 0.2 M and pH 4.0 at RT overnight to give [Cys^{29}–Tyr^{38}]-[Cys^{39}–Glu^{61}]-$CONH_2$ (Figure S3B). The product was purified by semi-preparative RP-HPLC to give 23.5 mg, 6.21 μmol (78.2 % yield). Peptide segments [Cys^{29}–Tyr^{38}]-[Cys^{39}–Glu^{61}]-$CONH_2$ (12.7 mg, 3.4 μmol) and [Gly^{1}–Ala^{28}]-$^{\alpha}$thioester (9.0 mg, 3.1 μmol) were dissolved in NCL buffer (Figure S3C) and incubated at pH 7.0 for 4.5 h to give [Gly^{1}–Ala^{28}]-[Cys^{29}–Tyr^{38}]-[Cys^{39}–Glu^{61}]-$CONH_2$ (Figure S3D). The product was purified by semi-preparative RP-HPLC to give 6.0 mg, 0.92 μmol (38.5% yield).

Desulfurization of [Gly^{1}–Ala^{28}]-[Cys^{29}–Tyr^{38}]-[Cys^{39}–Glu^{61}]-$CONH_2$ Desulfurization was performed according to the protocol by Murakami, M., et al.[3] Full-length peptide [Gly^{1}–Ala^{28}]-[Cys^{29}–Tyr^{38}]-[Cys^{39}–Glu^{61}]-$CONH_2$ (6.0 mg) was dissolved to a concentration of 0.25 μM in 6 M guanidine·HCl, 0.45 M TCEP·HCl, 0.3 M MESNa and 5 μM VA-044 in 0.2 M sodium phosphate buffer at pH 7.0. The reactions were incubated at RT for 8 h and pH was adjusted back to 7.0 every hour. Upon completion of the desulfurization reactions, the products were purified by semi-preparative RP-HPLC. Fractions containing the desulfurized [Gly^{1}–Glu^{61}]-$CONH_2$ were combined and lyophilized to give 2.8 mg, 0.43 μmol (47.1% yield) (Figure S3E).

Folding of [Gly^{1}–Glu^{61}]-$CONH_2$ The desulfurized peptide [Gly^{1}–Glu^{61}]-$CONH_2$ was dissolved in 6 M guanidine·HCl, 20 mM TCEP·HCl, 20 mM Tris, and 150 mM NaCl, pH 8.5, and was serially diluted from 6 M to 3 M to 1 M guanidine·HCl using the same buffer without guanidine·HCl. The peptide solution was desalted into 20 mM Tris-HCl, 150 mM NaCl, pH 8.5 using a HiTrap Desalting column (GE Healthcare, UK). The folded protein was concentrated using a 3 kDa concentrator to give 1.3 mg, 0.20 μmol (46.4% yield) D-GB1 (Figure S3F).

Circular dichroism (CD) spectroscopy of folded proteins

Circular dichroism spectra of D-affibody and D-GB1 were recorded on an Aviv model 202 instrument at 25 °C. A 1 mm path length cell was used. The proteins were prepared by dissolving 0.06 mg in 50 mM sodium sulfate and 5 mM Tris-HCl at pH 8.5. The molar ellipticity (θ in deg cm^2 $dmol^{-1}$) was calculated by $[\theta]_\lambda = \theta_{obs}$ x 1/(10 lcn), where θ_{obs} = observed ellipticity at λ, l = pathlength (cm), c = concentration of peptide (M), n = # of amino acids. The mirror image form of the L proteins showed the same optical rotation, but with the opposite sign, confirming the mirror image fold of the D-proteins (Figure S4).

Synthesis of biotinylated p53/MDM2 Inhibitor D-peptide

Biotinylated p53/MDM2 inhibitor D-peptide was synthesized using Fmoc SPPS. The N-terminal glycine residue was protected with Boc. After the peptide was synthesized, the Alloc (allyloxycarbonyl) protecting group was removed as described above. Biotin (0.5 mmol) dissolved in 0.2 M HBTU (0.95 eq) was activated with 1.4 eq DIEA then coupled for 25 min at RT. The peptide was cleaved and purified and described above.

Construction of plasmids for recombinant proteins

The gene for $^{25\text{-}109}$MDM2 was purchased from Addgene (pGEX-4T MDM2 WT, 16237).[5]

The pET SUMO-$^{25\text{-}109}$MDM2 was prepared using the Champion pET SUMO protein expression system (Invitrogen, CA). AccuPrime Taq DNA polymerase (Invitrogen, CA) was used to PCR amplify the DNA using the following primers:

5'-GAGACCCTGGTTAGACCAAAGC-3' (forward)

5'-TTATACTACCAAGTTCCTGTAGATCATGG-3' (reverse)

The PCR product was purified by the QIAquick PCR purification kit (Qiagen, Netherlands), then cloned into pET SUMO by an overnight ligation at 15 °C with 3 ng PCR product, 50 ng pET SUMO vector, and 1 µl T4 DNA ligase. 2 µl of the ligation product was transformed into One Shot Mach1-T1 competent cells and plated on 30 µg/mL kanamycin plates and incubated overnight at 37 °C. Colonies were grown in LB media containing 30 µg/mL kanamycin. The plasmid DNA was isolated using the Qiaprep spin miniprep kit (Qiagen, Netherlands).

Protein expression and purification

SUMO-$^{25\text{-}109}$ MDM2 was expressed in Rosetta (DE3) pLysS cells in 1L LB culture. His_6-SUMO-LF_N-DTA (C186S)-LPSTGG-His_5, His_6-SUMO-LF_N-LPSTGG-His_6, His_6-SUMO-LF_N-DTA (C186S), SrtA*-His_6, WT anthrax protective antigen (PA), and PA[F427H] were expressed in *E. coli* BL21 (DE3) cells at New England Regional Center of Excellence/Biodefense and Emerging Infectious Diseases (NERCE).

Approximately 40 g of cell pellet was resuspended in 100 ml of 50 mM Tris-HCl, 150 mM NaCl, pH 7.5 buffer containing 200 mg lysozyme, 4 mg Roche DNAase I, and 2 tablet of Roche protease inhibitor cocktail then sonicated for three times for 20 seconds. After sonication, the suspension was centrifuged at 17,000 rpm for 40 minutes. The lysate was loaded onto three 5 ml HisTrap FF crude Ni-NTA column (GE Healthcare, UK) and washed with 100 mL of 20 mM Tris-HCl pH 8.5, 150 mM NaCl, at pH 8.5 and 100 mL of 40 mM imidazole in 20 mM Tris-HCl pH 8.5, 500 mM NaCl. The protein was eluted from the column using 500 mM imidazole in 20 mM Tris-HCl pH 8.5, 500 mM NaCl. The eluted protein was buffer exchanged into 20 mM Tris-HCl pH 8.5, 150 mM NaCl using a HiPrep 26/10 Desalting column (GE Healthcare, UK). WT PA and PA[F427H] was overexpressed in the periplasm of *E. coli* BL21 (DE3) cells and purified by anion exchange chromatography.[6]

One-pot sortagging reaction using *Staphylococcus aureus* SrtA*

Enzyme-mediated ligation using Sortase A was utilized to ligate the peptide and protein cargo to LF_N-DTA-LPSTGG (for SDv's) or LF_N-LPSTGG (for Sv's), depending on the assay. We used *Staphylococcus aureus* $SrtA_{59\text{-}206}$ that was evolved (P94S/D160N/K196T) by Chen et al. (SrtA*).[7]

N-terminal small ubiquitin-like modifier (SUMO) was first cleaved from the protein substrate, His_6-SUMO-LF_N-DTA-LPSTGG-His_5 (or His_6-SUMO-LF_N-LPSTGG-His_6), using 1 µg SUMO protease per mg of protein substrate at RT for 30 minutes to give the native N-terminus of LF_N-DTA-LPSTGG-His_5 (or LF_N-LPSTGG-His_6).

The ligation reactions were performed on Ni-NTA agarose beads using SrtA*. Ni-NTA beads were washed three times with SrtA* buffer (10 mM $CaCl_2$, 50 mM Tris-HCl, 150 mM NaCl, pH 7.5). In one pot, 50 µM LF_N-DTA-LPSTGG-His_5 (or LF_N-LPSTGG-His_6), 5 µM SrtA*, and 100 to 500 µM G_5-cargo were incubated with Ni-NTA beads in SrtA* buffer for 30 min at RT nutating. After 30 min, the Ni-NTA beads containing any unreacted LF_N-DTA-LPSTGG-His_5, SrtA*-His_6, His_6-SUMO, and GG-His_6 were spun down at 4 °C to minimize the formation of the hydrolyzed LF_N-DTA-LPST (or LF_N-LPST) side product. The supernatant containing the

sortagged LF_N-DTA variant (SDv) or (sortagged LF_N; Sv) was collected. The beads were washed three times with 1 mL of 20 mM Tris-HCl, 150 mM NaCl, pH 7.5. Three buffer exchanges were performed into 20 mM Tris-HCl, 150 mM NaCl, pH 7.5 to remove the excess G_5-peptide. Size exclusion chromatography into 20 mM Tris-HCl, 150 mM NaCl, pH 7.5 was used to remove the excess G_5-protein. The purity of the ligated product was analyzed by LC-MS.

Translocation of SDv's and protein inhibition assay in CHO-K1 cells

The CHO-K1 cells were maintained in F-12K media supplemented with 10% (v/v) fetal bovine serum at 37 °C and 5% CO_2. The cells were plated at 3.0 x 10^4 per well in a 96-well plate one day prior to the assay. The SDv's were prepared in ten-fold serial dilutions in 50 μL, and added 50 μL of F-12K media containing 20 nM PA. The 100 μL samples were added to CHO-K1 cells and incubated for 30 minutes at 37 °C and 5% CO_2. (SDv4 and SDv4-biotin was incubated for 2 hours.) After incubation with SDv's, the cells were washed three times with PBS and then 100 μL leucine-free F-12K medium supplemented with 1 μCi/mL ^{3}H-leucine (Perkin Elmer, MA) was added and incubated for 1 h at 37 °C 5% CO_2. The cells were washed three times with PBS and suspended in 150 μL of scintillation fluid. ^{3}H-Leu incorporation into cellular proteins was measured to determine the inhibition of protein synthesis by LF_N-DTA. The scintillation counts from cells treated with only PA were used as control value for normalization. Each experiment was done in triplicate.

The data were plotted using Origin8 using Sigmoidal Boltzmann Fit using equation $y = A2 + \frac{A1-A2}{1+e^{\frac{x-x_0}{dx}}}$ and x_0 represents the $\log EC_{50}$ values.

Cytosolic protein extraction and whole cell lysate preparation for western blot

CHO-K1 cells were plated in 12-well plate. The cells were treated with 250 nM Sv1, 2, 4, and 5 in the presence of 40 nM PA overnight at 37 °C and 5% CO_2. After treatment, the cells were washed with PBS and lifted with 0.25% trypsin-EDTA for 5 minutes to remove surface bound proteins then washed twice with PBS. For cytosolic protein extraction, 1 x 10^6 cells were resuspended in 100 μL of 50 μg mL^{-1} digitonin in 75 mM NaCl, 1 mM NaH_2PO_4, 8 mM Na_2HPO_4, 250 mM sucrose supplemented with Roche protease inhibitor cocktail for 10 min on ice, and centrifuged for 5 minutes at 13,000 rpm. For whole cell lysate, cells were lysed in IP lysis buffer (25 mM Tris, 150 mM NaCl, 1% v/v NP-40, pH 7.5) supplemented with Roche protease inhibitor cocktail on ice for 30 minutes and centrifuged for 10 minutes at 13,000 rpm. Both supernatants were collected for blotting and analysis.

Western Blot of Sv1, 2, 4, and 5 translocated into CHO-K1 cells

Lysates were run on an SDS-PAGE gel then transferred onto a nitrocellulose membrane soaked in 48 mM Tris, 39 mM glycine, 0.0375% SDS, 20% methanol and using a TE 70 Semi-Dry Transfer Unit (GE). After transferring, the membrane was blocked at room temperature for 2 hours with LI-COR blocking buffer. The membrane was then incubated with anti-LF or anti-Erk1/2, and anti-Rab5 in TBST overnight at 4 ºC. The membrane was washed and incubated with the appropriate secondary antibodies in TBST for 1 h then imaged by the LI-COR Odyssey infrared imaging system.

Trypsin digestion of L- and D-Affibody

Digestion of 3 μg L- and D-affibody with 3 ng trypsin at 37 °C in 100 μL were monitored up to 23 hours. The digestion was monitored by LC-MS.

Pull down of Sv4-biotin in U-87 MG cells

U-87 MG cells were grown in T-75 flasks at a density of $5x10^6$ per flask. Cells were treated with 100 nM Sv3-biotin in the presence of 20 nM PA or PA[F427H] for 24 hours at 37 °C and 5% CO_2. After treatment, the cells were lifted with 0.25% trypsin-EDTA, washed three times, and lysed with 500 μL IP buffer for 10 min on ice. As a negative control, untreated cells. Lysates were incubated with 20 μL (42 μg) streptavidin-agarose beads (Sigma Aldrich) for 4 hours at 4 °C. The beads were washed three times with lysis buffer and once with lysis buffer without NP-40. The bound proteins were eluted in 1X Laemmli sample buffer (Bio-Rad), boiled for 10 min, and then analyzed by SDS-PAGE gel. Standard immunoblotting was performed using anti-MDM2 antibody and streptavidin-680.

Translocation of Sv4 and Sv4-biotin in U-87 MG or K562 cells

U-87 MG cells were grown in T-25 flasks at a density of $1x10^6$ per flask. K562 cells were treated in 24 well dishes at a density of 8.0 x 10^5 per well. Cells were treated with 150 nM of Sv1, Sv4, or Sv4-biotin in the presence of 20 nM PA or 20 nM PA[F427H] for 24 hours at 37 °C and 5% CO_2. As a positive control, cells were treated with 1 μM nutlin-3 for 24 h. After treatment, cells were lifted by trypsin and lysed with IP lysis buffer. The isolated lysates were subjected SDS-PAGE separation and transferred to a nitrocellulose blot as previously described. The membrane was immunoblotted with anti-LF, anti-MDM2, anti-p21, anti-p53, and anti-GAPDH overnight in TBST. The membrane was washed and incubated with the appropriate secondary antibody in TBST for 1 h and then imaged.

The western blot images were analyzed and quantified using the Image Studio program (LI-COR). Each band was normalized to the GAPDH loading control. The band intensity of each protein was set to one in the PA only control. Percent increase in amount of MDM2, p53, and p21 present in each lane compared to the PA only control was plotted in Figure S6.

Binding interaction between SUMO-$^{25\text{-}109}$MDM2 and 4 or Sv4

A competition binding assay was performed using a bilayer interferometry system (Octet RED96, ForteBio, CA) to determine the binding affinity of 4 (peptide) and Sv4 to SUMO-$^{25\text{-}109}$MDM2. Super streptavidin (SSA) sensors were soaked in 1X kinetics buffer (1X PBS containing 0.02% Tween-20, 0.1% BSA, pH 7.4) for 10 minutes at 30 °C. For immobilization, the wild-type $^{15\text{-}29}$p53 peptide was synthesized containing a biotin on the N-terminus (biotin-$^{15\text{-}29}$p53). The biotin-$^{15\text{-}29}$p53 peptide (1 μM in 1X kinetics buffer) was immobilized on the SSA sensor surface for 5 minutes at 30 °C and 1000 rpm. The surface was washed for 5 minutes with 1X kinetics buffer to establish a baseline. Two-fold dilutions of SUMO-$^{25\text{-}109}$MDM2 in 1X kinetics buffer were analyzed for binding over 5 minutes at 30 °C and 1000 rpm followed by dissociation with 1X kinetics buffer. A calibration curve was generated using GraphPad Prism 6 software using non-linear regression analysis (one-site, specific binding fit). The K_d was found to be 578 ± 170 nM.

For the competition assay, various concentrations of 4 (peptide) or Sv4 were incubated with 50 nM SUMO-$^{25\text{-}109}$MDM2 at room temperature for 30 minutes. Meanwhile, SSA sensors were soaked in 1X kinetics buffer for 10 minutes at 30 °C. The biotin-$^{15\text{-}29}$p53 peptide (1 μM) was immobilized on the SSA sensor surface then washed to establish a baseline, as previously described for the calibration curve. After immobilization, the association and dissociation of SUMO-$^{25\text{-}109}$MDM2 pre-incubated with 4 or Sv4 samples were analyzed at 30 °C and 1000 rpm

and using 1X kinetics buffer. Based on the binding (nm) values, the concentration of unbound MDM2 was interpolated for each sample using the calibration curve.

Non-linear regression analysis was performed using GraphPad Prism 6 software to determine the K_d value based on the equation: K_d = [peptide][MDM2]/[complex].[8] We used the following equation to generate fitted curves: $y = \frac{(b-x-K_d)+\sqrt{(b+x+K_d)^2-4(bx)}}{2}$; where y is [MDM2] in nM, x is [inhibitor] in nM, K_d is the dissociation constant, and b is y_{max}.

Table S1. Peptides used in this investigation

Sequence	Observed (Da)	Calculated (Da; monoisotopic)
**1:** (L)-G_5-AKFRPDSNVRG-$CONH_2$	1529.8 ± 0.1	1529.8
**2:** (D)-G_5-akfrpdsnvrg-$CONH_2$	1529.8 ± 0.1	1529.8
**3:** (D-cap) G_5-AKFRPDSNvrg-$CONH_2$	1529.8 ± 0.1	1529.8
**4:** (D) G_5-tawyanf(CF_3)ekllr	1865.0 ± 0.1	1864.9
**4-biotin:** (D) G_5-k(biotin)-$(GGs)_3$-tawyanf(CF_3)ekllr	2820.0 ± 0.1	2820.5
**biotin-$^{15\text{-}29}$p53:** (L) biotin-SQETFSDLWZLLPEN-$CONH_2$	2031.2 ± 0.1	2031.2

Table S2. Observed molecular masses of expressed protein constructs when analyzed by LC-MS

Protein	Observed MW (Da)	Calculated MW (Da; average)
SrtA*-His_6	19214.6 ± 0.4	19214.5
LF_N-DTA	52047.0 ± 0.4	52046.2
His_6-SUMO-LF_N-DTA-LPSTGG-His_5	66700.0 ± 0.4	66700.1
His_6-SUMO-LF_N-LPSTGG-His_6	45289.8 ± 0.4	45289.8
WT PA	83754.1 ± 0.4	83751.6
PA[F427H]	83742.4 ± 0.4	83738.3
SUMO-$^{25\text{-}109}$MDM2	23297.3 ± 0.4	23296.6
L-affibody	6925.8 ± 0.2	6925.6
L-GB1	6481.4 ± 0.2	6481.0

Table S3. List of variants

Variant	Sequence
SDv1	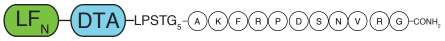
Sv1	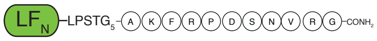
SDv2	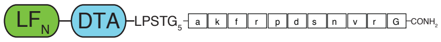
Sv2	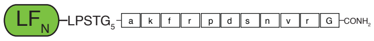
SDv3	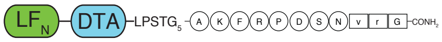
Sv3	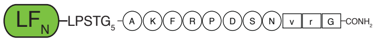
SDv4	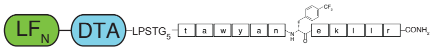
Sv4	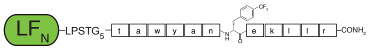
SDv4-biotin	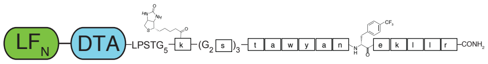
Sv4-biotin	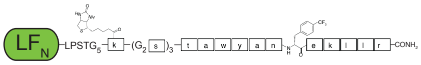
SDv5	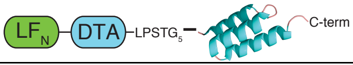
Sv5	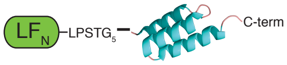
Sv5-alkyne	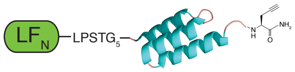
Sv5-biotin	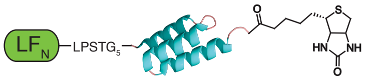
SDv6	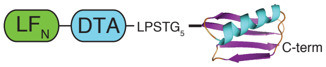

Table S4. Isolated yields of sortagging ligations from SrtA* reaction

Protein	Isolated Yield (%)
SDv1	61
Sv1	41
SDv2	66
Sv3	53
Sv4	76
Sv4-biotin	77
SDv5	14
Sv5	51
Sv5-alkyne	38
SDv6	29

Table S5. EC50 values of 30-minute protein synthesis inhibition assay. The errors represent fitting errors from Sigmoidal Boltzmann Fit. (* represents 2 h assay)

Protein	EC50 (pM)
LF_N-DTA	21 ± 3
SDv1	37 ± 5
SDv2	24 ± 3
SDv5	20 ± 4
Sv5-alkyne	51 ± 15
SDv6	33 ± 6
LF_N-DTA*	2.0 ± 0.6
SDv4*	3.2 ± 0.7
SDv4-biotin*	7.1 ± 2.5

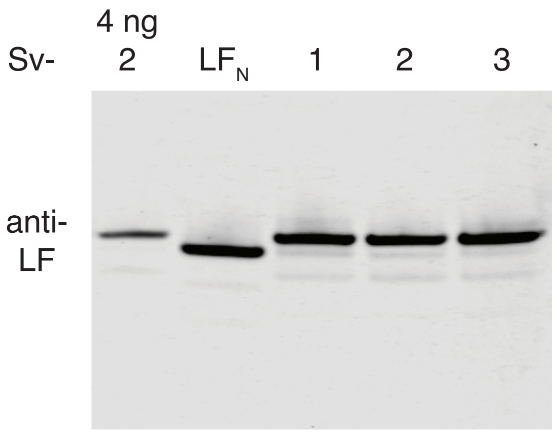

Figure S1. Immunoblot of media from CHO-K1 cells treated with Sv1-3 (Figure 2c). Serum-containing F12K with 250 nM wild-type LF_N or Sv1-3 and 40 nM PA was removed after overnight treatment with CHO-K1 cells. The stability of the LF_N variants in media was analyzed by immunostaining with anti-LF.

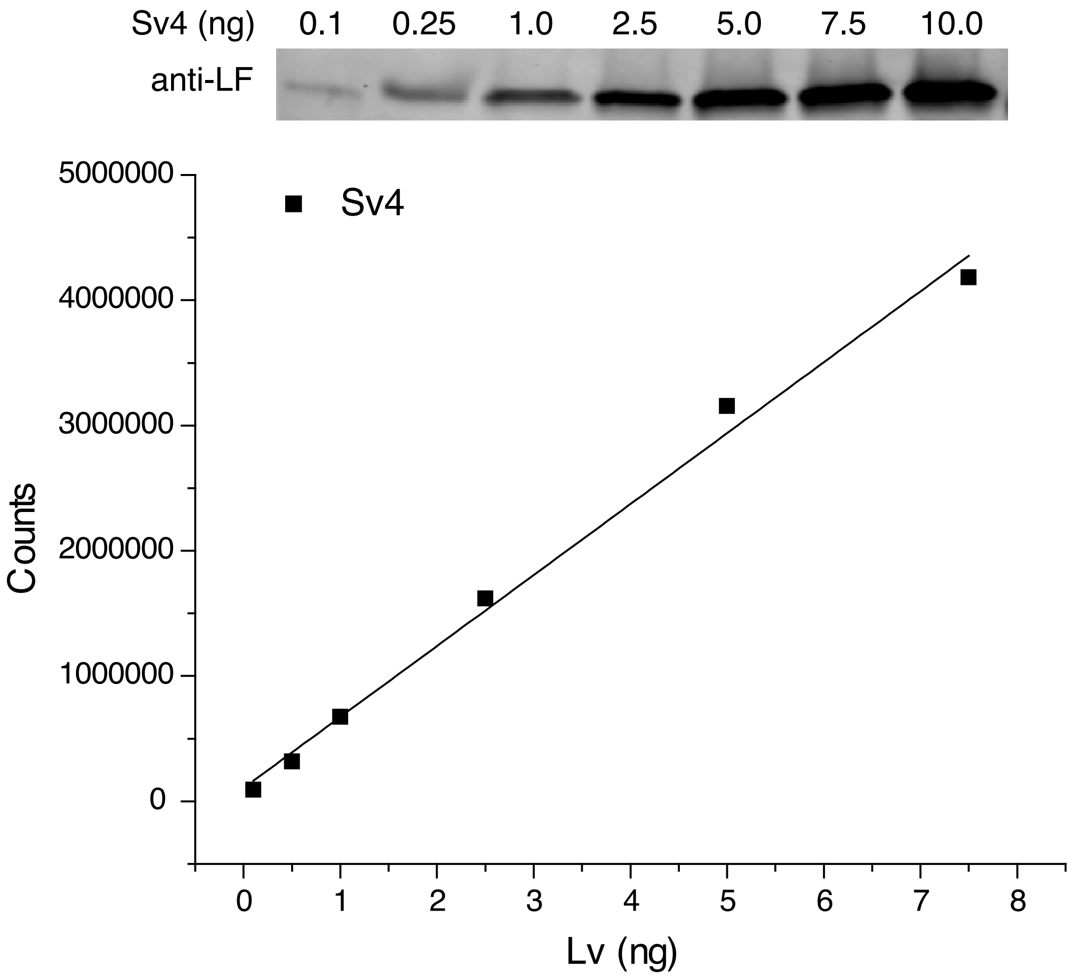

Figure S2. Linear relationship between Sv4 band intensity and the amount of protein loaded (ng): y = 566000x + 107000, $R^2 = 0.993$). The signal intensity of each band was compared to 0.1 – 10 ng of pure Sv4 protein loaded on the gel and immunoblotted with anti-LF.

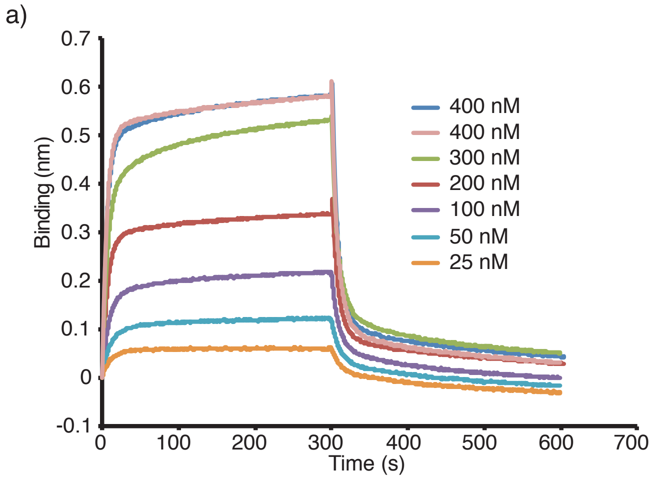

Figure S3. Calibration curve for the interaction between immobilized biotin-$^{15\text{-}29}$p53 and SUMO-$^{25\text{-}109}$MDM2 determined using Octet RED96 bilayer interferometry. a) Association and dissociation curves of various concentrations of SUMO-$^{25\text{-}109}$MDM2 with biotin-$^{15\text{-}29}$p53 immobilized to super streptavidin sensors. b) A calibration curve was generated using GraphPad Prism 6 software using non-linear regression analysis (one-site, specific binding fit). The K_d was found to be 578 ± 170 nM.

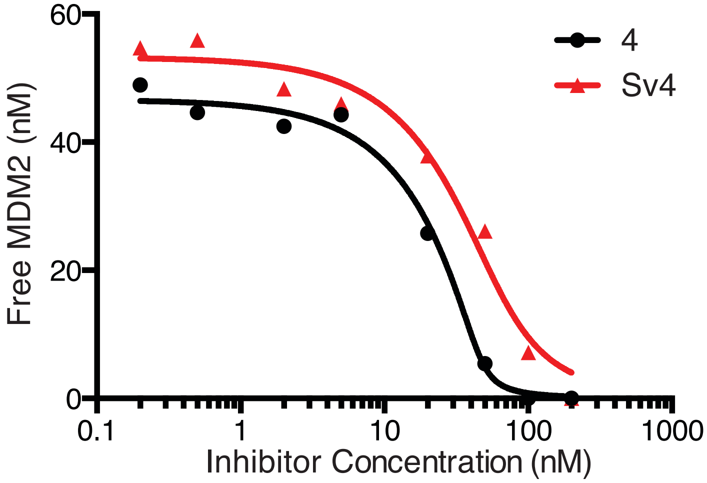

Figure S4. Binding interaction between SUMO-$^{25\text{-}109}$MDM2 and 4 or Sv4 based on a competition binding assay at 30 °C. Non-linear regression analysis was used to generate fitted curves and determine the K_d values based on the equation: K_d = [peptide][MDM2]/[complex]. Non-linear regression analysis was performed using GraphPad Prism 6 software to determine the K_d value based on the equation: K_d = [peptide][MDM2]/[complex].[8] We used the following equation to generate fitted curves: $y = \frac{(b-x-K_d)+\sqrt{(b+x+K_d)^2-4(bx)}}{2}$; where y is [MDM2] in nM, x is [inhibitor] in nM, K_d is the dissociation constant, and b is y_{max}. The K_d values calculated for 4 and Sv4 were 1.0 ± 0.7 nM and 12.3 ± 4.3 nM, respectively.

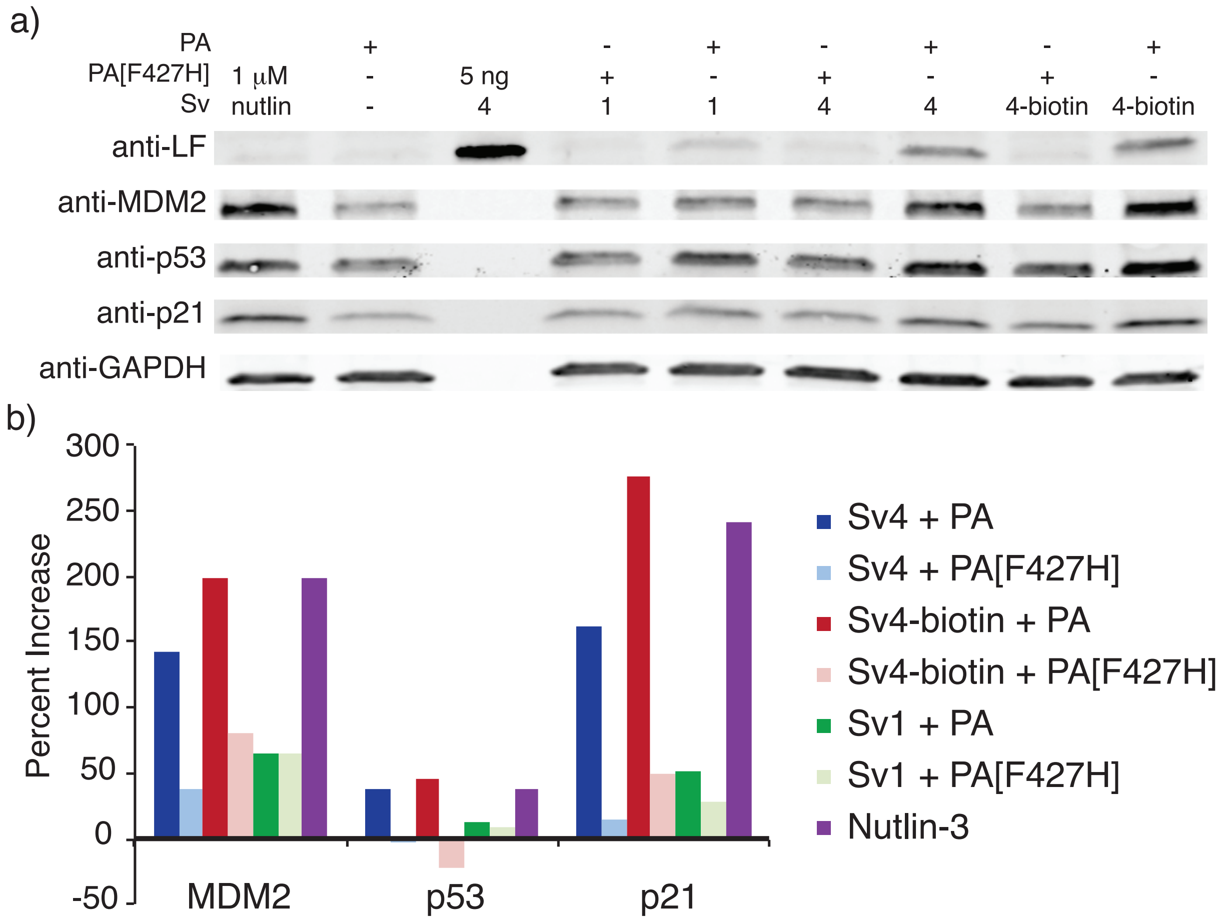

Figure S5. Quantification of MDM2, p53, and p21 protein levels for U-87 MG cells treated with Sv1, Sv4, or Sv4-biotin in the presence of PA or PA[F427H] based on western blot data from Figure 3d. All levels were normalized to the anti-GAPDH loading control and the PA only condition.

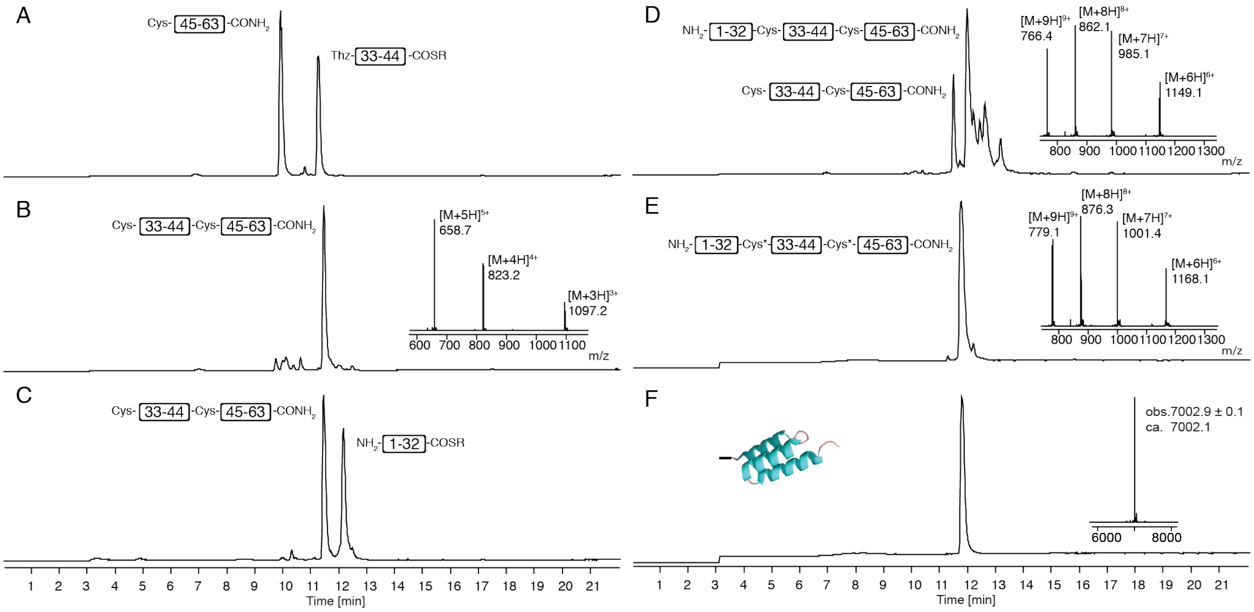

Figure S6. LC-MS characterization of D-affibody synthesis

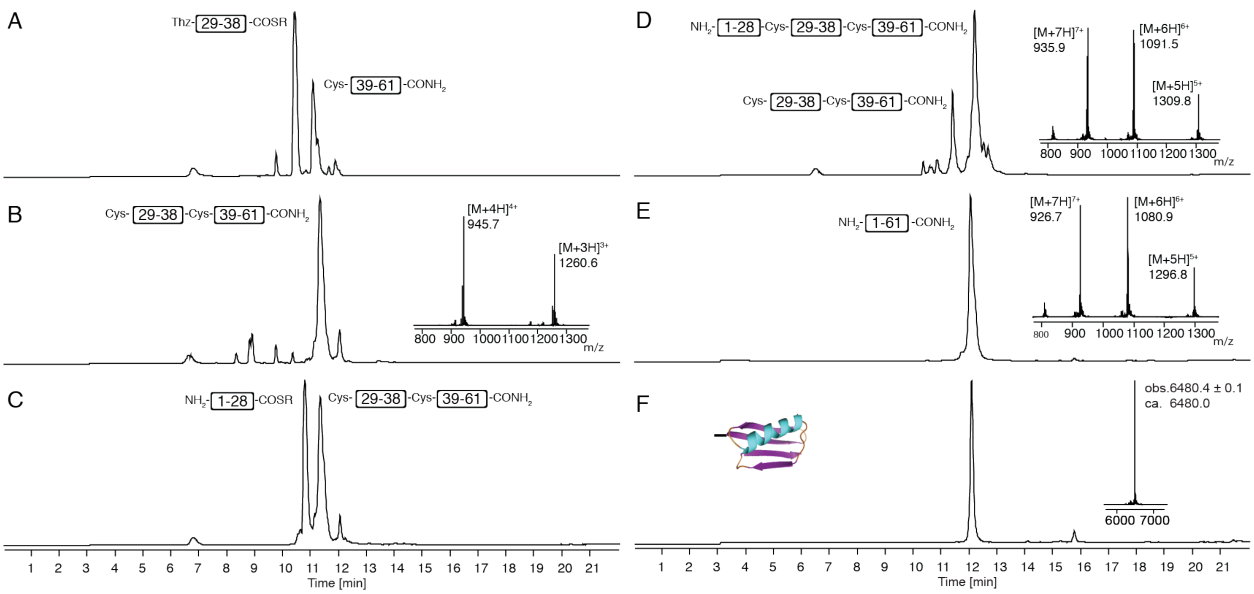

Figure S7. LC-MS characterization of D-GB1 synthesis

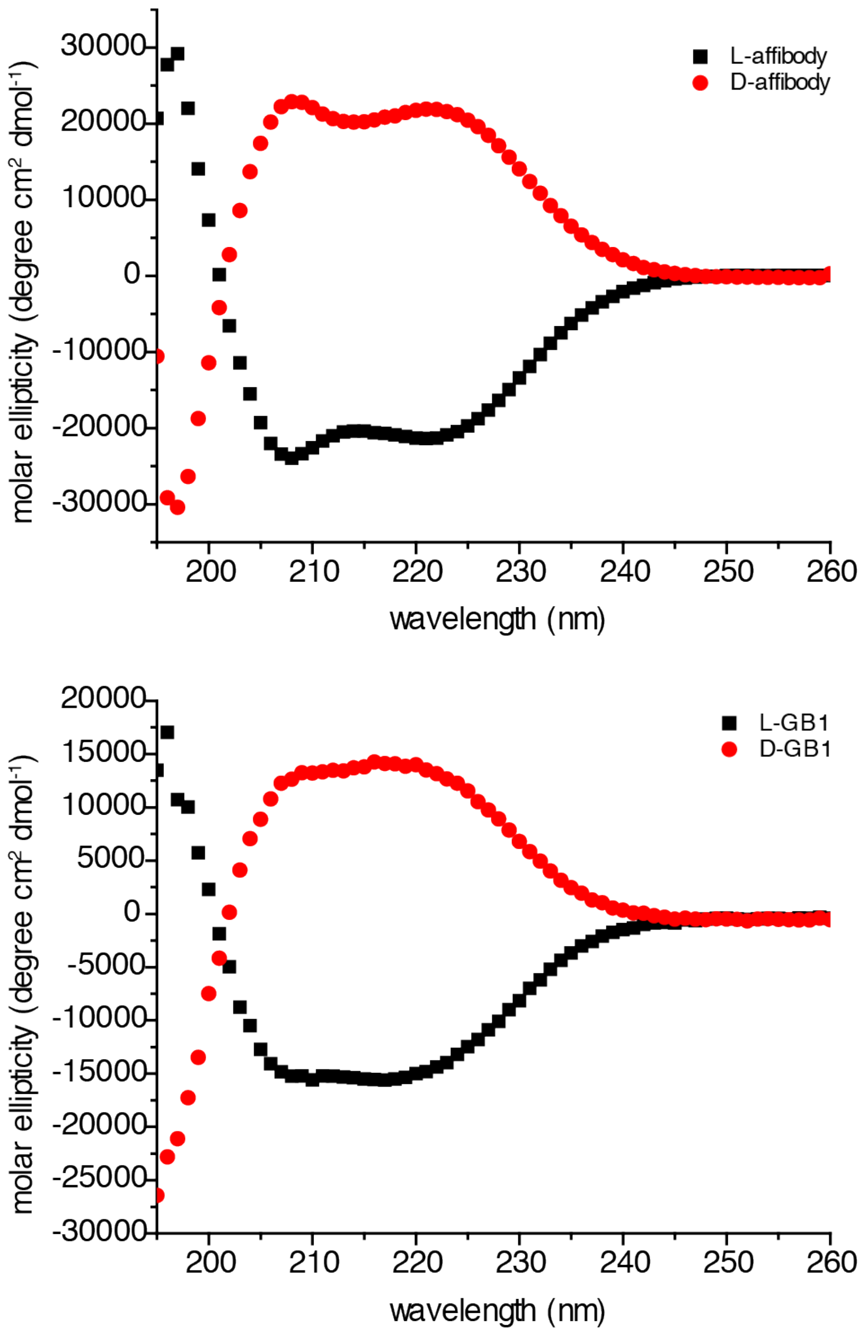

Figure S8. CD of L- and D-affibody and L- and D-GB1.

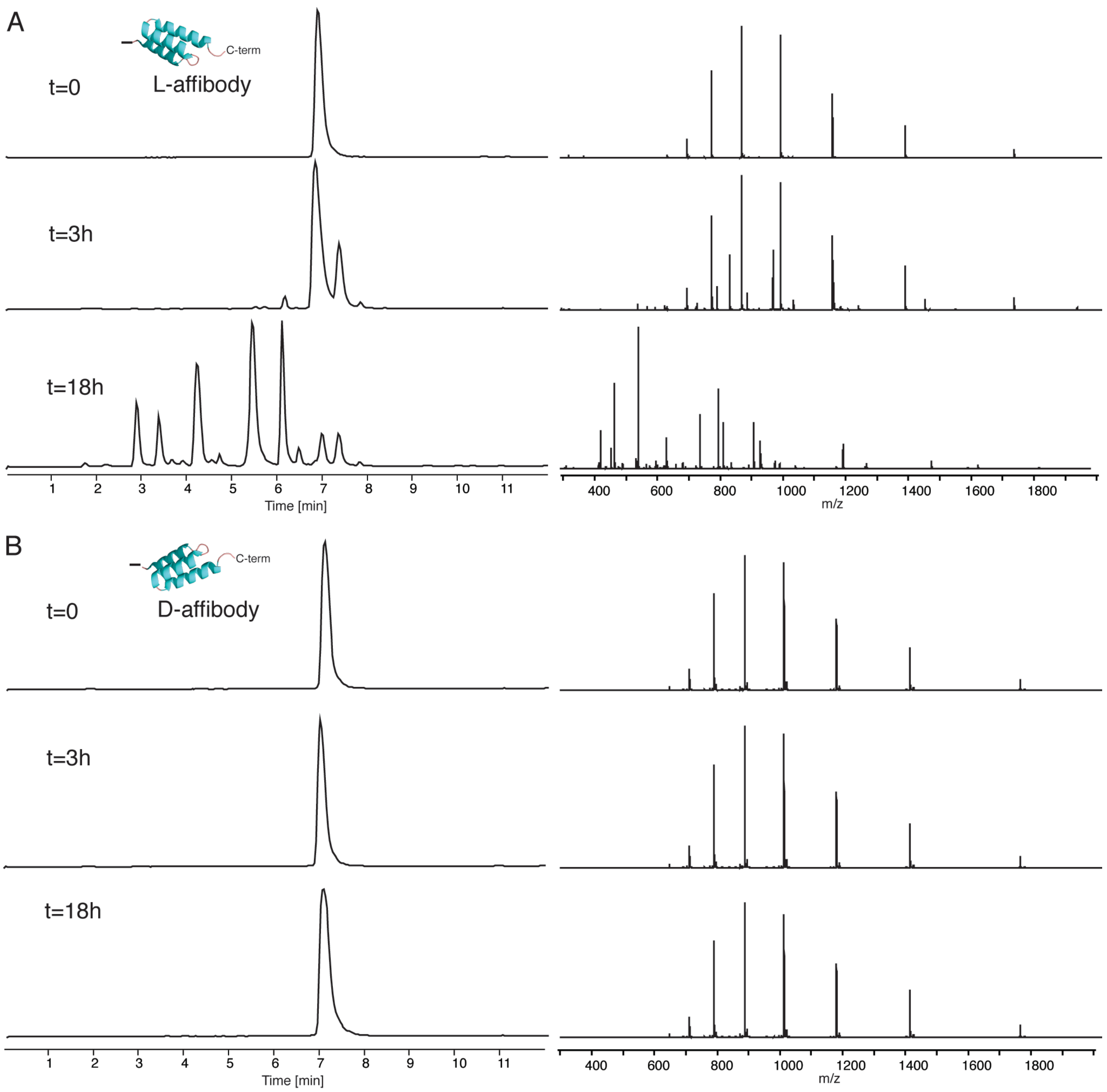
A
C-term
L-affibody
t=0
t=3h
t=18h
1 2 3 4 5 6 7 8 9 10 11
Time [min]
400 600 800 1000 1200 1400 1600 1800
m/z
B
C-term
D-affibody
t=0
t=3h
t=18h
1 2 3 4 5 6 7 8 9 10 11
Time [min]
400 600 800 1000 1200 1400 1600 1800
m/z

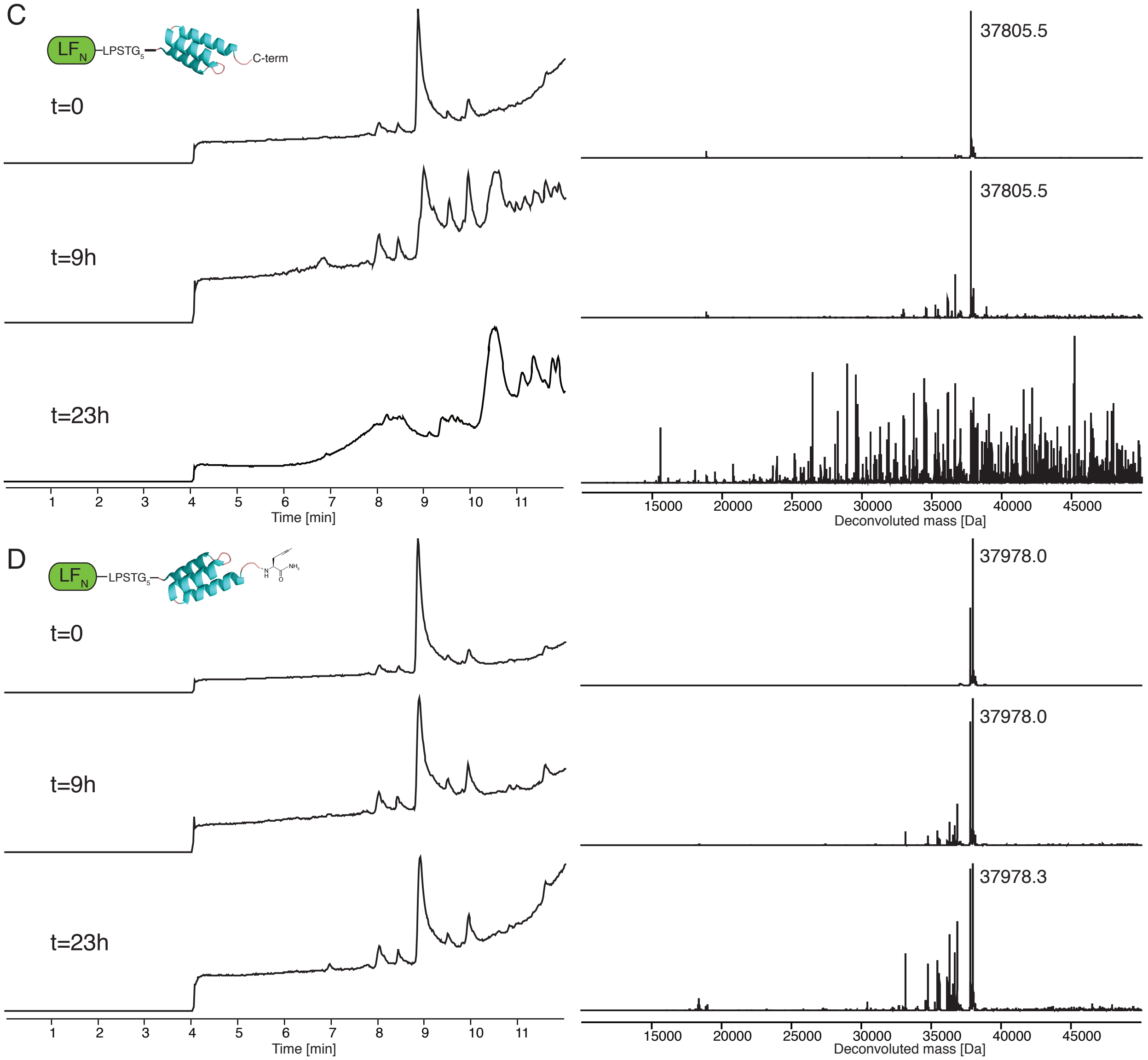

Figure S9. Trypsin digestion of L-affibody (A) and D-affibody (B) at t=0, 3, and 18 h and Sv5-L (C) and Sv5-alkyne at t=0, 9, and 23 h. For A-D, the left panel corresponds to the total ion current (TIC). For A and B, the right panel corresponds to the mass spectra of the major peak area and for C and D, the right panel corresponds to the deconvoluted mass of the major peak area.

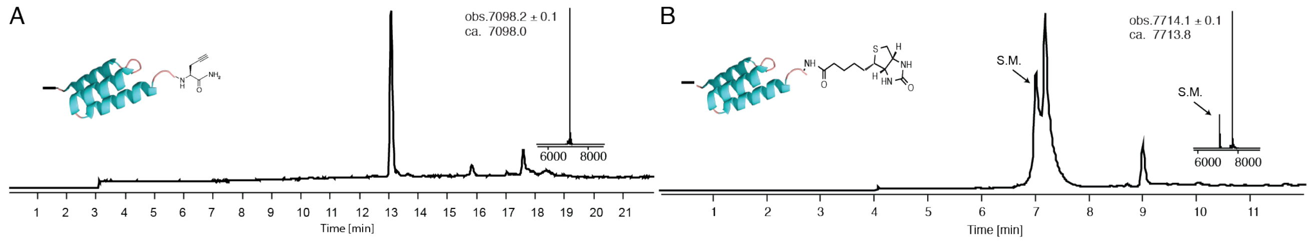

Figure S10. LC-MS characterization of D-affibody-alkyne (a) and D-affibody-biotin (b)

LC-MS Appendix:

SDv1

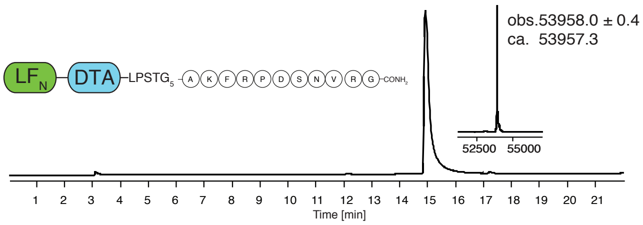

Sv1

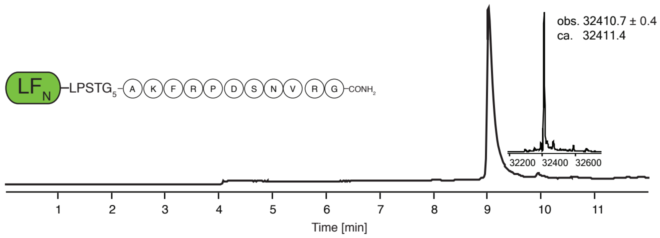

SDv2

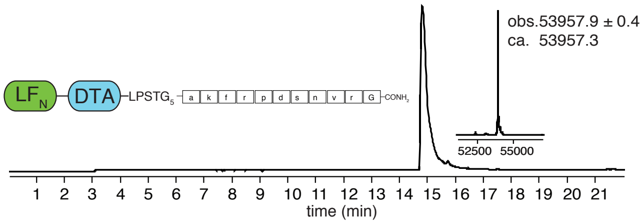

Sv2

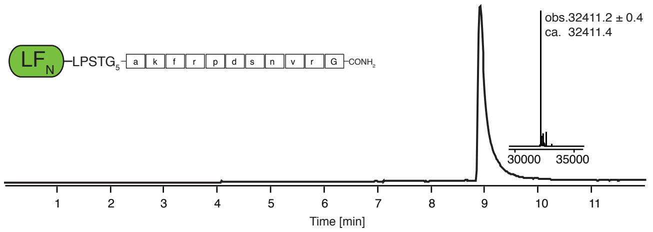

Sv3

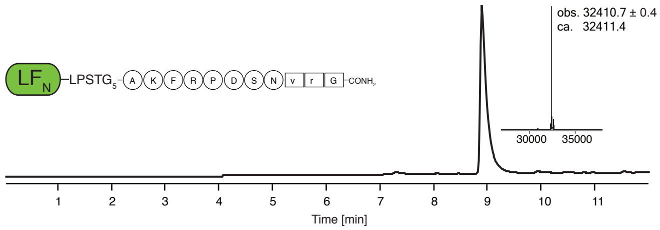

SDv4

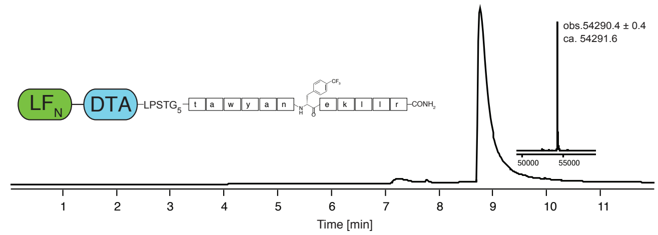

Sv4

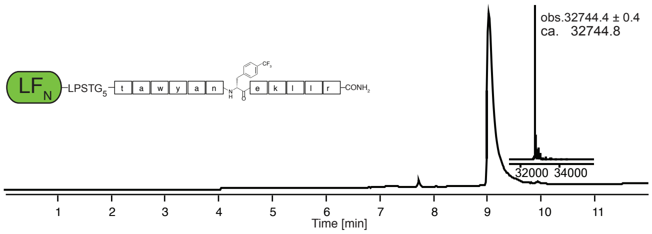

SDv4-biotin

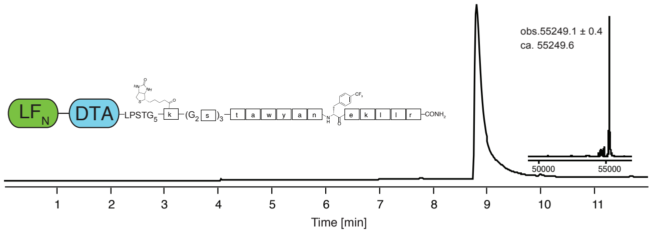

Sv4-biotin

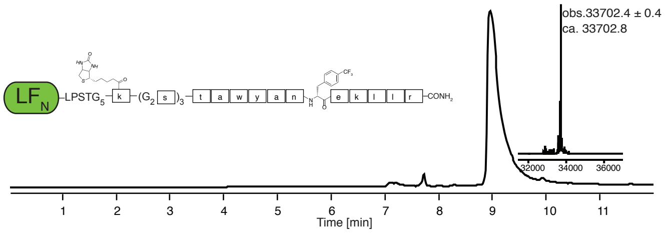

SDv5

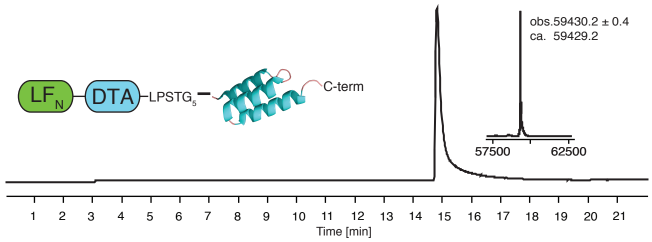

Sv5

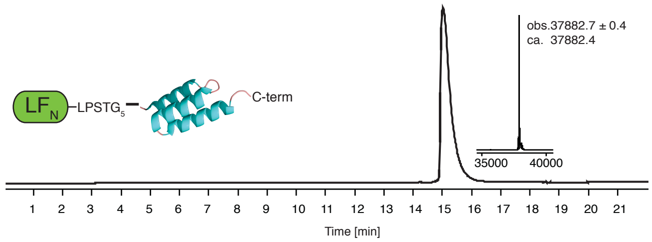

Sv5-alkyne

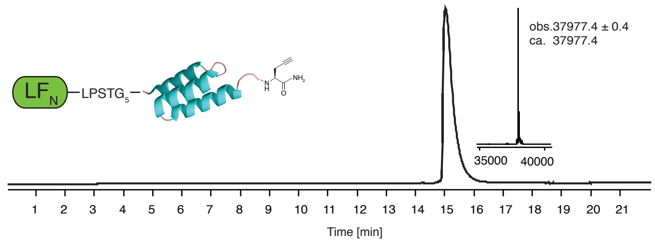

Sv5-biotin

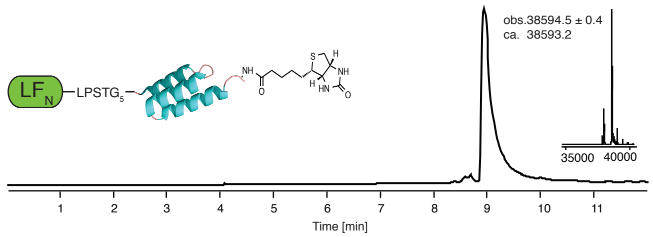

SDv6

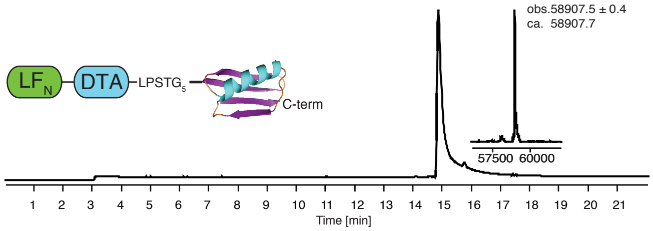

References

[1] M. Schnolzer, P. Alewood, A. Jones, D. Alewood, S. B. H. Kent, *Int. J. Pept. Protein Res.* **1992**, *40*, 180-193.

[2] T. M. Hackeng, J. H. Griffin, P. E. Dawson, *Proceedings of the National Academy of Sciences of the United States of America* **1999**, *96*, 10068-10073.

[3] (a) Q. Wan, S. J. Danishefsky, *Angew. Chem.-Int. Edit.* **2007**, *46*, 9248-9252; (b) M. Murakami, R. Okamoto, M. Izumi, Y. Kajihara, *Angew. Chem.-Int. Edit.* **2012**, *51*, 3567-3572.

[4] (a) M. D. Simon, P. L. Heider, A. Adamo, A. A. Vinogradov, S. K. Mong, X. Y. Li, T. Berger, R. L. Policarpo, C. Zhang, Y. K. Zou, X. L. Liao, A. M. Spokoyny, K. F. Jensen, B. L. Pentelute, *Chembiochem* **2014**, *15*, 713-720; (b) S. K. Mong, A. A. Vinogradov, M. D. Simon, B. L. Pentelute, *Chembiochem* **2014**, *15*, 721-733.

[5] B. H. P. Zhou, Y. Liao, W. Y. Xia, Y. Y. Zou, B. Spohn, M. C. Hung, *Nat. Cell Biol.* **2001**, *3*, 973-982.

[6] C. J. Miller, J. L. Elliott, R. J. Collier, *Biochemistry* **1999**, *38*, 10432-10441.

[7] (a) S. K. Mazmanian, G. Liu, T. T. Hung, O. Schneewind, *Science* **1999**, *285*, 760-763; (b) I. Chen, B. M. Dorr, D. R. Liu, *Proc. Natl. Acad. Sci. U. S. A.* **2011**, *108*, 11399-11404; (c) M. W. Popp, J. M. Antos, G. M. Grotenbreg, E. Spooner, H. L. Ploegh, *Nat. Chem. Biol.* **2007**, *3*, 707-708.

[8] C. Zhan, L. Zhao, X. Wei, X. Wu, X. Chen, W. Yuan, W.-Y. Lu, M. Pazgier, W. Lu, *J. Med. Chem.* **2012**, *55*, 6237-6241.
